# Evaluation of dental caries detection with quantitative light-induced fluorescence in comparison to different field of view devices

**DOI:** 10.1038/s41598-022-10126-x

**Published:** 2022-04-12

**Authors:** Song Hee Oh, Jin-Young Choi, Seong-Hun Kim

**Affiliations:** 1 Department of Oral and Maxillofacial Radiology, Graduate School of Dentistry, Kyung Hee University, Seoul, Korea; 2 Department of Orthodontics, Graduate School of Dentistry, Kyung Hee University, Seoul, Korea

**Keywords:** Anatomy, Diseases, Health care

## Abstract

This study evaluated dental caries detection ability between the Qraycam and Qraypen on the same dental caries lesions. A total of 178teeth from 61patients were imaged using Qraypen C®(QC) and Qraycam Pro®(QP) devices and evaluated using analysis software (QA2). Occlusal, secondary, and proximal dental caries were evaluated and scored according to International Caries Detection and Assessment System(ICDAS II) and X-ray criteria. Bland–Altman plots were used to compare quantitative light-induced fluorescence(QLF) parameters obtained from the different QLF devices. Sensitivity, specificity, and area under the receiver operating characteristic curve(AUROC) were calculated. The ΔF_aver._ of the QLF-parameters showed that the mean difference between the two different QLF devices was close to zero and that the ± 5 error value was included in the mean ± 1.96SD range for the detection of dental caries. The accuracies for diagnosing occlusal dental caries were 0.83–0.96 and 0.81–0.82 and the accuracies for diagnosing proximal dental caries were 0.52–0.62 and 0.52–0.71 for the QC and QP devices, respectively. In conclusion, the ΔF_aver._ obtained from the QP showed diagnostic value mainly for screening of demineralized teeth. For teeth selected through screening, the depth of the lesion must be precisely evaluated using additional QP and radiographic imaging.

## Introduction

Over the past two decades, there has been a paradigm shift in the early detection of carious lesions without cavities and nonsurgical treatment of such lesions before cavity formation^1–4^. These changes require the early detection and evaluation of lesions; thus, accurate diagnosis is essential^5^.

In general, visual inspection is the simplest and most commonly used method for detecting caries in the clinical setting; however, this technique is limited in that it can only detect decay directly on the tooth surface. Due to the anatomical structure of the lesion, such detection is possible only after the lesion has progressed significantly to the dentin^6–8^. Thus, visual inspection for detecting noncavitary lesions varies widely in both sensitivity (0.2–0.96) and specificity (0.50–1.00) and is inconsistent across examiners as a diagnostic tool^9,10^. Radiography, which has long been used as a traditional diagnostic method, is now considered unsuitable for reliable diagnosis owing to its low sensitivity (0.14–0.38) and high specificity (0.59–0.90) in the detection of early carious lesions^9,10^. Additionally, the American Dental Association (ADA) discourages the use of routine radiographic examinations for screening purposes due to the inherent risks of radiation exposure^11^. Currently, the options available for detecting dental caries beyond visible assessment are limited.

In this context, various adjunctive diagnostic techniques, such as quantitative light-induced fluorescence (QLF), optical fiber-transmitted illumination using visible light, DIAGNOdent using laser light, and electrical conductivity measurement using electrical current, have been investigated^10,12^. The QLF technique can quantitatively evaluate minute changes in teeth based on autofluorescence that occurs when irradiated with 405 nm visible blue light^13,14^. The loss of fluorescence detected in teeth was highly correlated with the loss of minerals within the lesion, which can be used to effectively detect and monitor minute changes in demineralization/remineralization in early carious lesions without cavities^15,16^. Red fluorescence is derived from porphyrin-induced metabolites produced by oral microorganisms and is emitted by dental caries, dental plaques, and calculi^17,18^. This technique showed an intraclass correlation coefficient (ICC) of 0.96 and intra- and inter-examiner agreements of 0.93 and 0.92, respectively. Therefore, in the diagnosis of early carious lesions, QLF can reduce the probability of a diagnostic mismatch if only visual inspection and radiographic examinations are employed^10^.

Various types of devices using the QLF technology have been developed. Among them, the Qraypen C® (QC, AIOBIO, Seoul, Republic of Korea), with a small field of view (FOV), was introduced in 2018. This device comprises blue (emits 405-nm peak wavelength) and white LEDs, an inductor filter, and a 1/3-inch progressive complementary metal–oxide–semiconductor (CMOS) image sensor (HD 720p). The Qraycam Pro® (QP, AIOBIO, Seoul, Republic of Korea) with a larger FOV, introduced in 2018, is a third-generation QLF device that comprises a set of LEDs (in the same configuration as QLF-D), an Inspektor glass filter (with the same filter as in the QLF-D device), and a CMOS image sensor (FHD 1080p).

These two QLF devices are used differently for different imaging purposes, owing to their different FOV. The QC provides a large FOV and is useful for screening the overall dental condition in the oral cavity. However, the image quality of the QC is relatively deteriorated because it is placed outside the oral cavity. In contrast, the QP device is placed in the oral cavity, making it suitable for individual teeth and proximal imaging, enabling detailed evaluation.

Therefore, the purpose of this study was to evaluate the clinical applicability of QC as a screening tool to detect dental caries through quantitative evaluation through fluorescence loss (ΔF) and red fluorescence (ΔR) parameters compared to the QP, which could be evaluated relatively precisely for the same dental carious lesion.

## Results

This study analyzed 178 teeth. Table 1 shows the mean and standard deviation of the QLF parameters for teeth diagnosed with 130 occlusal dental caries, 27 secondary dental caries, and 21 proximal dental caries.

**Table 1:** Average and standard deviation of QLF parameters according to dental caries score.

Score	Number of teeth	QC				QP			
Occlusal dental caries		|ΔF_max_ |	|ΔF_aver._|	ΔR_aver_	ΔR_max_	|ΔF_max_ |	|ΔF_aver._|	ΔR_aver_	ΔR_max_
1	56	36.4 ± 11.8	11.3 ± 2.2	21.1 ± 10.7	25.4 ± 25.8	29.1 ± 10.3	12.2 ± 2.9	10.8 ± 13.8	12.7 ± 22.1
2	62	61.2 ± 12.6	16.0 ± 3.1	32.1 ± 9.5	80.7 ± 53.7	47.7 ± 11.9	17.0 ± 4.1	27.4 ± 15.7	49.2 ± 47.2
3	12	84.8 ± 8.2	28.3 ± 5.8	69.7 ± 37.5	313.6 ± 197.9	65.7 ± 4.7	28.0 ± 8.6	55.2 ± 23.5	170.3 ± 122.9
**Secondary dental caries**									
1	5	26.8 ± 8.9	10.8 ± 1.6	26.6 ± 2.3	44.2 ± 11.1	30.7 ± 7.9	14.4 ± 3.3	28.2 ± 6.8	34.0 ± 29.7
2	22	48.7 ± 11.5	15.0 ± 3.1	32.4 ± 4.6	86.0 ± 40.8	43.5 ± 8.5	17.9 ± 2.3	35.6 ± 9.2	76.9 ± 34.2
**Proximal dental caries**									
1	8	21.8 ± 9.5	9.7 ± 2.7	17.5 ± 10.4	14.0 ± 18.5	23.0 ± 6.8	11.7 ± 3.1	12.1 ± 12.5	9.8 ± 17.2
2	6	31.8 ± 11.8	13.0 ± 2.7	28.3 ± 5.4	37.5 ± 20.8	29.2 ± 9.6	14.9 ± 4.4	30.7 ± 15.2	45.3 ± 24.5
3	7	21.4 ± 8.7	9.7 ± 2.3	20.6 ± 13.8	31.7 ± 22.9	21.6 ± 12.3	12.1 ± 6.9	24.9 ± 24.1	41.9 ± 43.1

Quantitative light-induced fluorescence (QLF); Qraycam Pro® (QP); Qraypen C® (QC).

**Figure 1:**
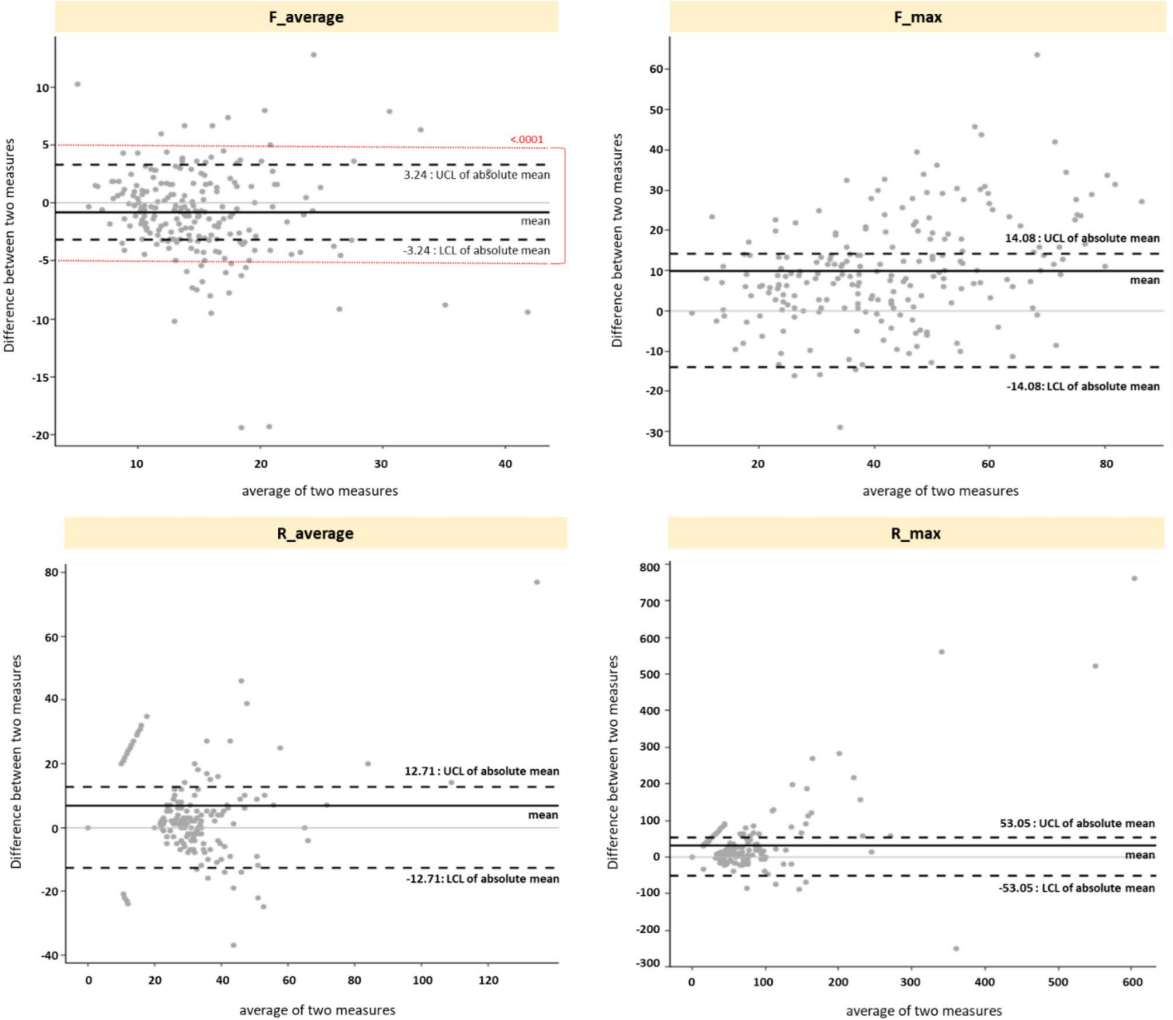
Agreements between the fluorescence parameters obtained from different devices (Bland–Altman plot).

We performed a Bland–Altman plot analysis to confirm the concordance between the fluorescence parameters of the two different devices using the confidence interval as the deviation ± 5 value of the error range provided by the manufacturer. Consequently, the ΔF_aver._ of the QLF parameters showed a mean difference between the two different QLF devices of close to zero and a ± 5 error value within the mean ± 1.96 SD range. Moreover, the probability of the ΔF_aver._ was within the confidence interval of 87.2% (Fig. 1).

**Figure 2:**
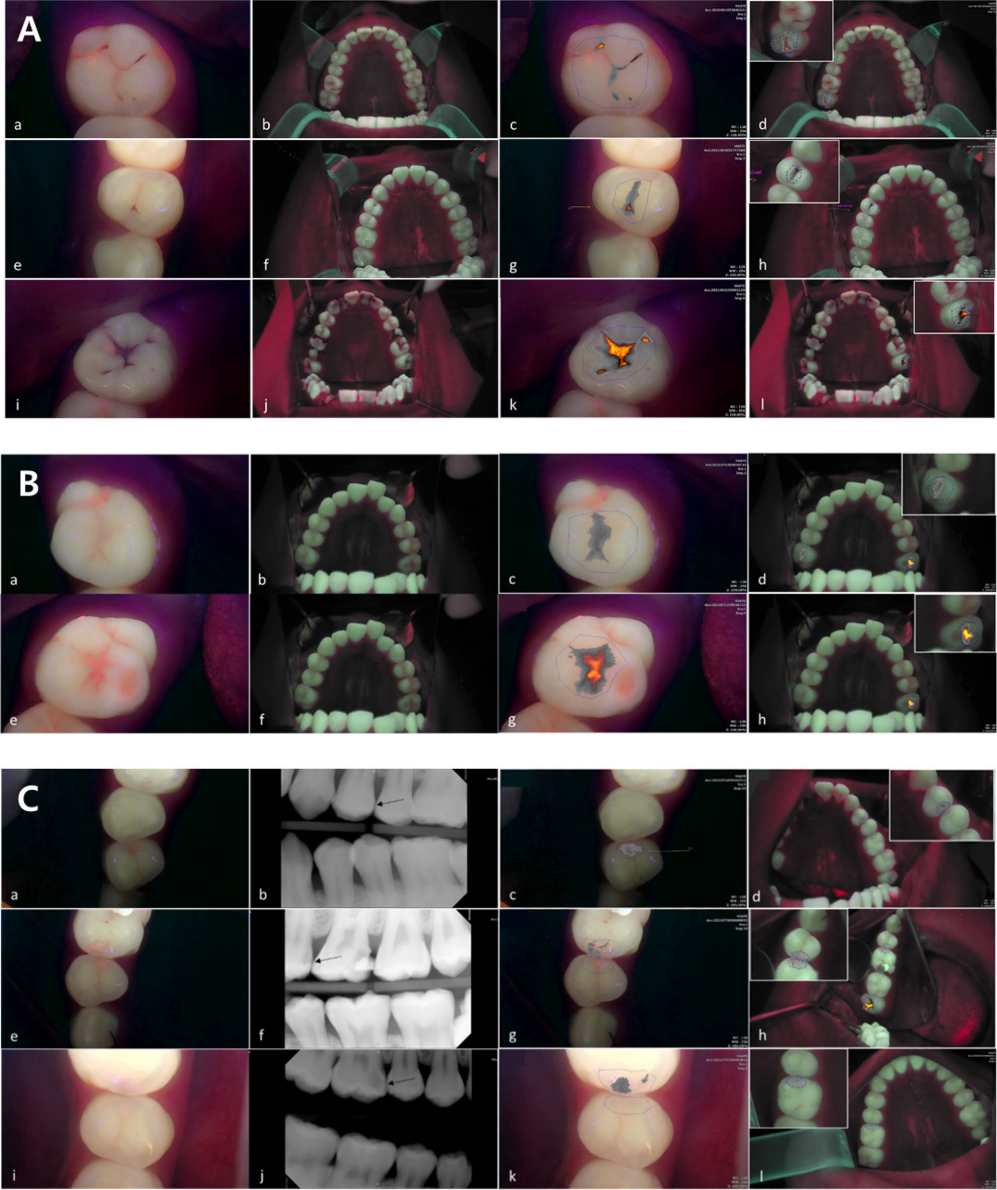
(**A**) Occlusal dental caries according to International Caries Detection and Assessment System (ICDAS) II criteria: (a–d) Score 1 (first visual change in the enamel) on the maxillary right second molar (#17). (e–h) Score 2 (distinct visual change in the enamel when viewed wet) on the maxillary right first premolar (#14). Score 3 (localized enamel breakdown) on the maxillary left second molar (#27). (a, e, i) Fluorescence image from the Qraypen C. (b, f, j) Fluorescence image from the Qraycam Pro. (c, g, k) Quantitative analysis of the Qraypen C image using QA2; (d, h, l) Quantitative analysis of the Qraycam Pro image using QA2. (**B**) Secondary dental caries according to the ICDAS II criteria: (a–d) Score 1 (first visual change in the enamel) on the maxillary left second molar (#27). (e–h) Score 2 (distinct visual change in the enamel when viewed wet) on maxillary left second molar (#27). (a, e) Fluorescence image from the Qraypen C. (b, f) Fluorescence image from the Qraycam Pro. (c, g) Quantitative analysis of the Qraypen C image using QA2. (d, h) Quantitative analysis of the Qraycam Pro image using QA2. (**C**) Proximal dental caries according to X-ray criteria: (a–d) Score 1 (radiolucency visible in the enamel) on the maxillary left first premolar (#24, arrow). (e–h) Score 2 (radiolucency restricted to the outer third of the dentin) on the maxillary left second premolar (#25, arrow). Score 3 (radiolucency extending to the middle third of the dentin) on the maxillary right first molar (#16, arrow). (a, e, i) Fluorescence image from the Qraypen C. (b, f, j) Bitewing radiograph. (c, g, k) Quantitative analysis of the Qraypen C image using QA2. (d, h, l) Quantitative analysis of the Qraycam Pro image using QA2.

Evaluation of the cut-off values and validity of the QLF parameters for diagnosing occlusal dental caries revealed an accuracy of 0.83–0.96, and an area under the receiver operating characteristic curve (AUROC) of 0.92–0.99 for the QC and 0.81–82 and 0.87–0.94, respectively, for the QP. Both devices showed a high diagnostic accuracy for dental caries. In particular, all parameter values showed higher accuracy in the diagnostic threshold for discriminating the degree of caries requiring treatment (D2) than in the diagnostic threshold for discriminating early dental caries (D1) (Table 2, Fig. 2A).

**Table 2:** Cut-off values and validity of QLF parameters for detecting occlusal dental caries.

Diagnostic thresholds	QLF parameters		Cut-off value	Sensitivity	Specificity	Accuracy	AUROC
D1	QC	|ΔF_aver._|	12.90	0.89	0.75	0.83	0.92
	QP	|ΔF_aver._|	13.40	0.91	0.68	0.81	0.87
D2	QC	|ΔF_aver._|	21.40	1.00	0.96	0.96	0.99
	QP	|ΔF_aver._|	18.20	0.92	0.81	0.82	0.94

Quantitative light-induced fluorescence (QLF), Qraycam Pro® (QP), Qraypen C® (QC).

Evaluation of the cut-off values and validity of the QLF parameters for diagnosing secondary dental caries showed an accuracy of 0.75 and AUROC of 0.90 for the QC and 0.89 and 0.82, respectively, for the QP (Table 3, Fig. 2B).

**Table 3:** Cut-off values and validity of QLF parameters for detecting secondary dental caries.

Diagnostic thresholds	QLF parameters		Cut-off value	Sensitivity	Specificity	Accuracy	AUROC
D1	QC	|ΔF_aver._|	13.10	0.74	0.80	0.75	0.90
	QP	|ΔF_aver._|	13.90	0.96	0.60	0.89	0.82

Quantitative light-induced fluorescence (QLF); Qraycam Pro® (QP); Qraypen C® (QC).

Evaluation of the cut-off values and validity of the QLF parameters for diagnosing proximal dental caries revealed an accuracy of 0.52–0.62, and an AUROC of 0.60–0.67 for the QC and 0.52–0.71 and 0.56–0.64, respectively, for the QP (Table 4, Fig. 2C).

**Table 4:** Cut-off values and validity of QLF parameters for detecting proximal dental caries.

Diagnostic thresholds	QLF parameters		Cut-off value	Sensitivity	Specificity	Accuracy	AUROC
D1	QC	|ΔF_aver._|	10.40	0.62	0.62	0.62	0.67
	QP	|ΔF_aver._|	13.60	0.38	0.75	0.52	0.56
D2	QC	|ΔF_aver._|	14.30	0.00	0.79	0.52	0.60
	QP	|ΔF_aver._|	11.00	0.57	0.79	0.71	0.64

Quantitative light-induced fluorescence (QLF); Qraycam Pro® (QP); Qraypen C® (QC).

## Discussion

The comparisons of the two QLF devices in this study showed significant agreement between ΔF_aver._ for the four QLF parameters used to detect dental caries. Previous studies have also demonstrated the usefulness of the QLF parameter ΔF for detecting early occlusal carious lesions^19^. As mentioned previously, the degree of fluorescence loss detected in the tooth was highly correlated with mineral loss within the lesion, which enables lesion diagnosis by effectively detecting and monitoring minute changes in demineralization/remineralization in early carious lesions without cavities^15,16^. However, one outstanding question is the clinical usefulness of diagnosing early dental caries by evaluating only enamel demineralization. Another study argued that long-existing white spot lesions did not show any more demineralization effects and should be considered when diagnosing early dental caries. Because long-standing white spot lesions appear to be stable, it may be important for clinicians to carefully determine whether the lesion is active or arrested^20^. In this context, the results showing a higher degree of agreement between the two devices for dental caries (D2), which are considered to require treatment in this study, were meaningful.

Only the QLF parameter ΔF_aver._ showed significant agreement among the four QLF parameters analyzed in the present study, although the same sites were examined in both QC and QP images. This difference was attributed to the size (pixels) of the object photographed by the two devices. In general, the demineralization area of the occlusal surface is shown as a red or yellow area on QC images taken from a close distance, with finely divided pixels. In the QP image taken from a long distance, the pixels were displayed as black instead of a red or yellow area because the pixels could not be distinguished and, instead, were displayed together. Accordingly, our results demonstrated that QC images showing red/yellow regions had large ΔF_max_ values, while the QP images did not show the corresponding value and concomitantly showed low-intensity values.

Previous studies using the QLF technique mainly focused on red fluorescence for mature and pathogenic plaques^21,22^. However, Lee et al. first reported, through an in vitro experiment, that the red fluorescence observed in mature pathogenic plaques is also present in occlusal carious lesions^23^. Demineralized early enamel carious lesions contain fewer minerals and higher amounts of organic matter and water than healthy enamel ^24,25^. This can lead to the formation of porous pathways that connect the inside and outside of the lesion. These results indicated that the QLF technique can be used to evaluate the activity and extent of microorganisms inside carious enamel lesions, suggesting that the ΔR value can be a useful parameter for evaluating such lesions. In particular, when diagnosing secondary caries using the QP, the ΔR value may be a more reliable parameter than the ΔF value. In the case of secondary caries, it is more meaningful to evaluate the activity of mature microorganisms deposited around the restoration than the degree of enamel demineralization when diagnosing early caries. However, this study did not determine the inter-device agreement or diagnostic accuracy for ΔR values for the detection of dental caries.

We observed a low correlation in proximal caries diagnosis between the two QLF devices. In general, diagnosing proximal caries is challenging due to the anatomy of the lesions, as visual detection of these caries is limited because 75% of proximal lesions are in contact sites, with the rest beneath them^15,26^. Proximal lesions are detected only when dentin is affected or the marginal ridges become cavitated^27^. Thus, the number of proximal caries may be underestimated when using only visual examination. Although attempts have been made to diagnose proximal caries using the QLF technique, this method is also limited due to the anatomical location of the lesion. These sites produce less fluorescence because the lesion blocks the excitation light from the device as well as the backscattered fluorescence from the dentine. This results in reduced fluorescence, making it difficult to detect fluorescence in proximal dental caries^15^. The present study evaluated proximal caries by capturing fluorescence images of the occlusal surface. This indirect evaluation, as in previous evaluations of proximal caries^26,27^, was limited in its ability to confirm the relationship between the lesion depth and fluorescence parameters. However, another study showed that QLF technology can be used as a screening tool to detect proximal dental caries at the dentin level before radiographic examination^28,29^.

The in vivo repeatability and reproducibility of the QLF are excellent for the quantification of dental surface caries and this device has been applied in several clinical studies^30^. However, standardized conditions should be used to obtain images of consistent quality. In this study, the QC and QP images were obtained using the same process by a single clinician with more than 3 years of QLF imaging experience. Plaques and external stains that could fluoresce and complicate the analysis of QLF images were also removed before the examination. In addition, the QLF images were acquired in the same room, and the image conditions were kept consistent by reducing the ambient lighting as much as possible. Using these standardized conditions, the image acquisition process likely had a minimal impact on the results of this study.

A limitation of this study was the absence of a gold standard method such as histological analysis. Previous studies used histological analysis to calculate the precise lesion depth to reconfirm the validity of QLF parameters and provide clear evidence for the diagnosis of early dental caries^31–33^; However, the current study used the International Caries Detection and Assessment System (ICDAS-II) to validate carious lesions, biasing the key aspect of validation. The correlation between ICDAS and histology decreased from 0.782 (when SM was used for histological validation) to 0.511 (when microradiography with a contrast solution was used for histological validation)^34^. Dental plaque and stains remaining on the tooth surface even after thorough cleaning before QLF imaging may have affected the results of this study. However, in vitro experiments are limited because they do not accurately reflect the actual clinical setting. This study was meaningful in that it considered the actual clinical process. Another limitation was that the reliability of the experimental results may be reduced because of the small number of proximal or secondary caries samples that were analyzed.

In conclusion, the mean value of F (ΔF_aver._) obtained from the QC device showed diagnostic value mainly for the screening demineralized teeth. For teeth selected through screening, the depth of the lesion must be precisely evaluated using the QP device and radiographic imaging.

## Methods

### Sample selection

**Figure 3:**
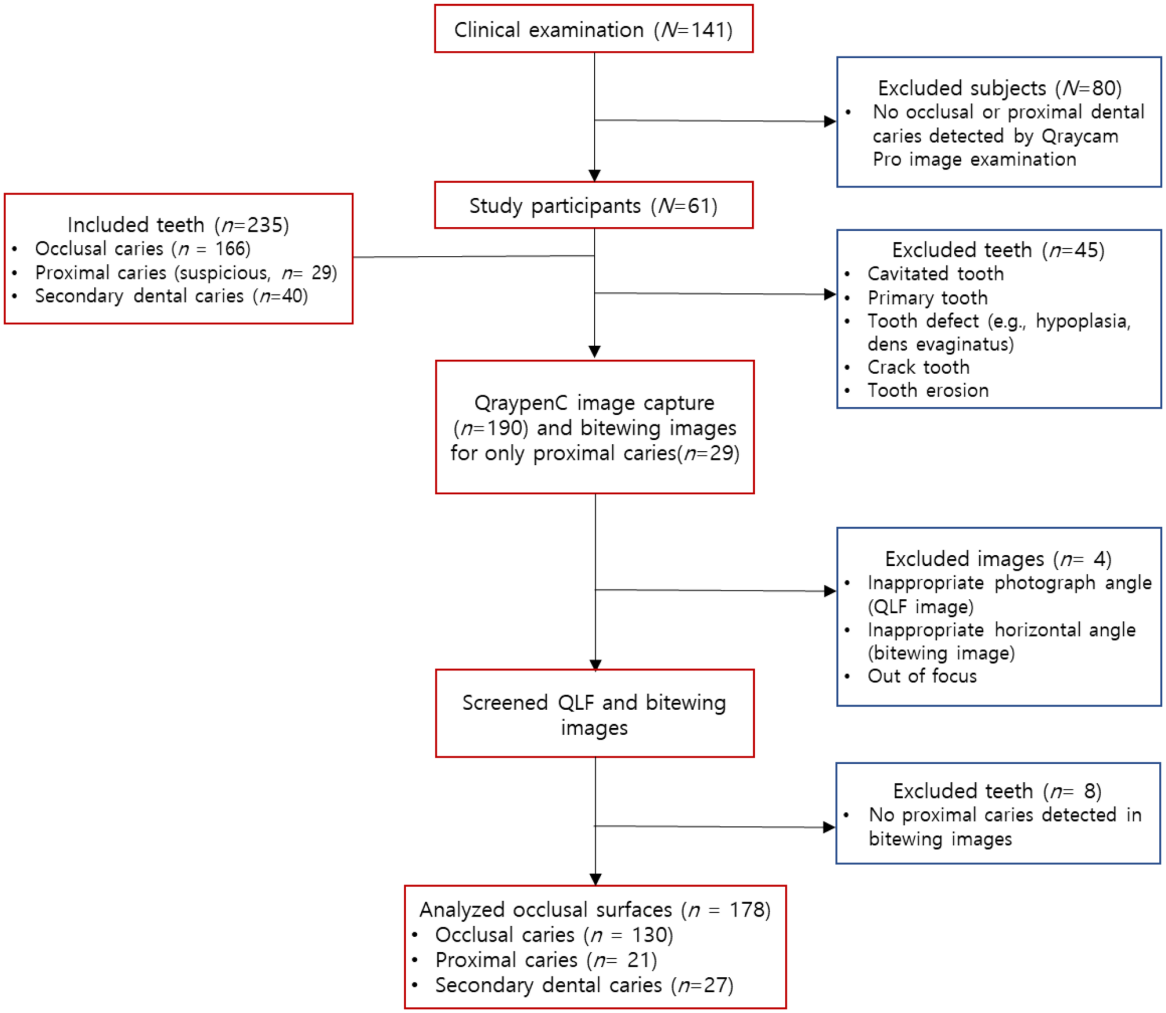
Flow diagram of the inclusion and exclusion for the diagnosis of dental caries (N = number of subjects, n = number of teeth).

This study was approved by the institutional review board of Kyung Hee University Dental Hospital (IRB No. KH-DT21019) and followed the tenets of the Declaration of Helsinki. Clinical data were collected from patients who visited Kyung Hee Dental Healthcare Center between April 2019 and August 2021. A total of 141 participants provided informed consent to participate and were initially enrolled in the study. After excluding 80 patients with no occlusal or proximal dental caries on QP examination, this study included 235 teeth from 61 patients. Among them, 166 teeth had occlusal caries, 29 had suspected proximal caries, and 40 had secondary caries. A total of 45 teeth were excluded following clinical examination for cavitated teeth, enamel hypoplasia, dens evaginatus, cracked teeth, and teeth with erosion, which could have affected the study results. Finally, this study included 178 teeth from 61 patients (Fig. 3). Among the 61 participants (35.6 ± 15.1 years), 29 were men (32.1 ± 12.0 years) and 32 were women (38.8 ± 17.0 years).

### Acquisition of QLF images

**Figure 4:**
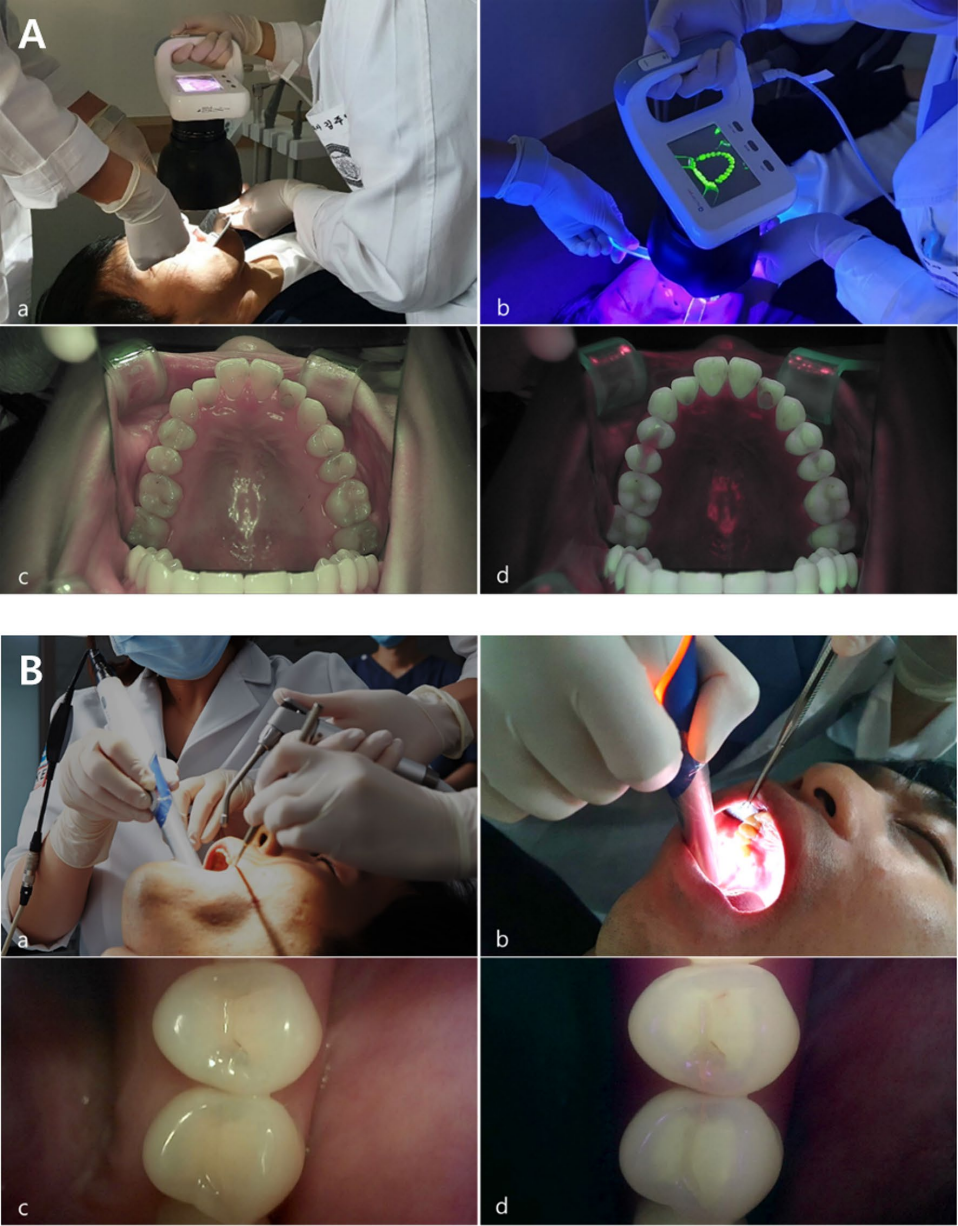

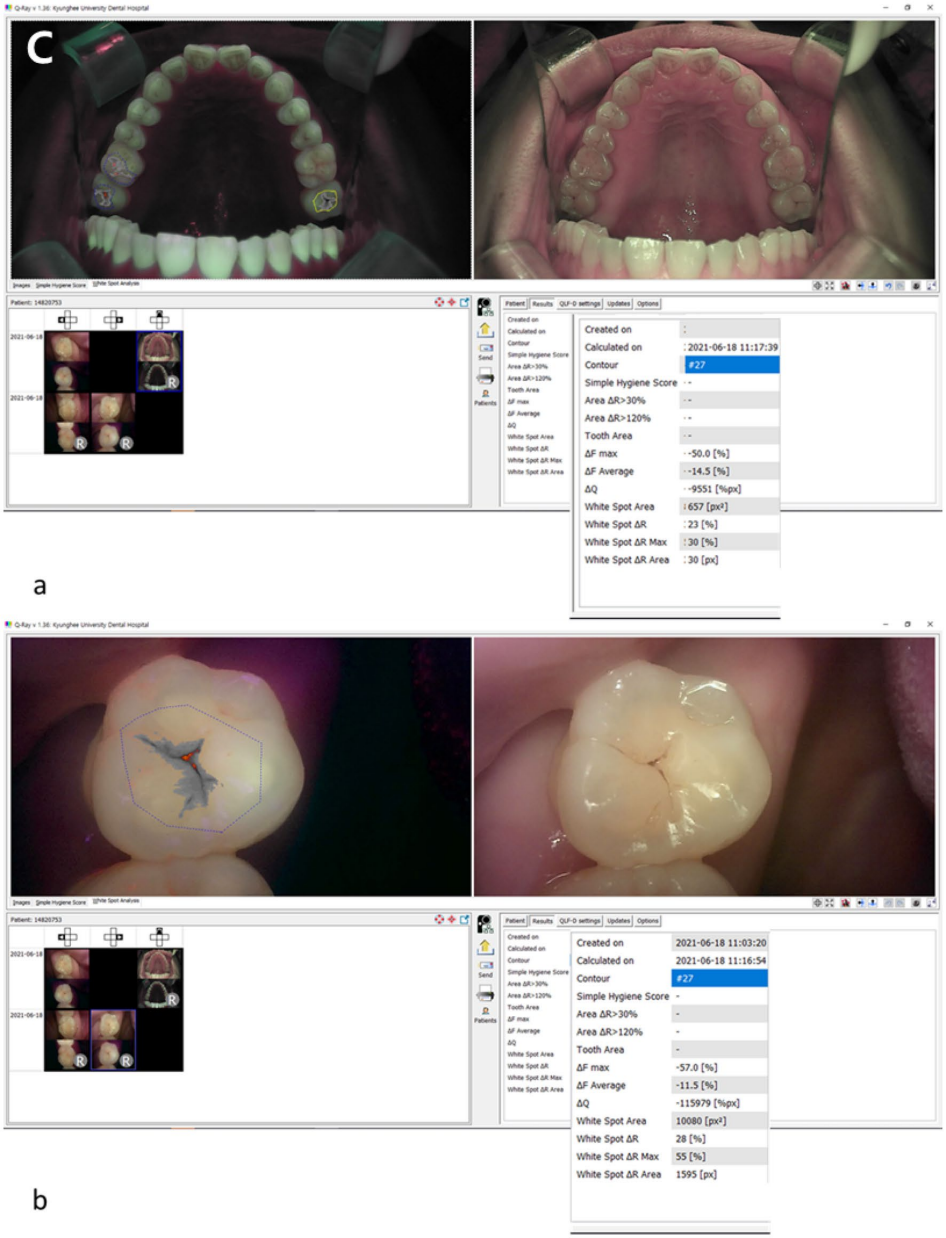
(**A**) White-light and fluorescent images of the occlusal surface of the maxilla or mandible captured using a Qraycam Pro® (QP, AIOBIO, Seoul, Republic of Korea). (a, b) A trained examiner using a Quantitative light-induced fluorescence (QLF) system in a dark room to maintain the image quality. This device is equipped with a metal tube that blocks external light to prevent contamination of the fluorescent image. (c) White-light image (d) Fluorescent image. (**B**) (a, b) QLF images of the occlusal surface of the examined tooth captured using a Qraypen C® (QC, AIOBIO, Seoul, Republic of Korea). (c) White-light image (d) Fluorescent image. (**C**) QA2 version 1.25 (Inspektor Research system BV, Amsterdam, The Netherlands). QA2 provides the QLF parameters (ΔF_max_, ΔF_aver._, ΔR_max_, ΔR_aver._) of the area of interest (AOI); (a, b) Quantitative analyses of QP and QC images, respectively.

White-light and fluorescence images were captured by a trained examiner using the QP and QC devices in a dark room to maintain image quality. The teeth were brushed with a toothbrush and gauze before QLF imaging to remove dental plaque or debris that could affect the results. The QLF images were captured with a QP (FOV, field of view (W × H × D, mm): 155 × 124 × 103, resolution: 1920 × 1080) using the “occlusal” imaging mode. This device was equipped with a metal tube and plastic shield to block external light and prevent interference with the fluorescence image. Using an intraoral photo mirror, the QP was placed vertically on the maxillary or mandibular occlusal surface containing the tooth to be analyzed and a mirror image was taken (Fig. 4A). The QC (field of view [FOV]: 5–45, resolution: 1280 × 720) was placed perpendicular to the occlusal surface of the corresponding tooth, and images were taken directly (Fig. 4B).

### Bitewing radiography

After the QLF images were obtained, bitewing radiographs were obtained only for the 29 teeth suspected of having proximal caries. Bitewing images were obtained using a digital sensor (Kodak RVG, Carestream Dental, NY, USA) and an X-ray unit (Asahi Roentgen Industry, Kyoto, Japan) at exposures of 60 kV and 7 mA, with an average exposure length of 0.63 s.

### Scoring of white QLF and bitewing images

The QLF and bitewing images were evaluated by an oral maxillofacial radiologist with > 10 years of experience. Occlusal and secondary dental caries were graded according to the ICDAS II criteria^35^, while proximal caries were graded according to X-ray criteria^36^. For lesions that were difficult to distinguish, a lower grade was assigned and the criteria were applied conservatively (Table 5).

**Table 5:** Criteria for the evaluation for dental caries.

Score	Occlusal and secondary dental caries according to ICDAS^a^ II criteria	Proximal dental caries according to X-ray criteria
1	First visual change in the enamel	Radiolucency visible in the enamel
2	Distinct visual change in the enamel when viewed wet	Radiolucency in the dentin but restricted to the outer third of the dentin
3	Localized enamel breakdown (without clinical visual signs of dentinal involvement)	Radiolucency extending to the middle 1/3 of the dentin

^a^Quantitative light-induced fluorescence (QLF); International Caries Detection and Assessment System (ICDAS II).

### Quantitative analysis of QLF images

Owing to the difference in fluorescence image size between the two devices, patches were drawn around the same area to be analyzed in each image and set as the region of interest (ROI). The fluorescence intensity of the ROI was quantified using two fluorescence parameters. The parameters ΔF and ΔR were measured as percentages using QA2 analysis software, version 1.25 (Inspektor Research Systems BV, Amsterdam, The Netherlands) (Fig. 4C). The ΔF value indicated the percentage decrease in fluorescence in the carious area relative to that of the normal enamel, which reflected changes in the mineral composition of the enamel. ΔR represented the increase in red fluorescence intensity due to porphyrins generated by bacterial metabolism as a percentage relative to that of normal enamel, which reflected the activity of microorganisms in the oral cavity.

### Statistical analysis

To evaluate inter-observer reproducibility, 20 cases were randomly selected, and another oral and maxillofacial radiologist with > 10 years of experience analyzed the QLF and bitewing images. To evaluate intra-observer reproducibility, 20 randomly selected cases were further analyzed after 2 weeks. The resultant intraclass correlation coefficient (ICC, 0.84–0.91; *P* < 0.001; kappa value, 0.85) and interobserver coefficient (ICC, 0.75–0.85; *P* < 0.001; kappa value, 0.80) indicated high reliability. Bland–Altman plots were used to compare the fluorescence parameters obtained from two different QLF devices. Receiver operating characteristic curves were plotted between the two devices to verify the validity of the fluorescence parameters for the diagnosis of dental caries. The sensitivity, specificity, and AUROC were calculated at two critical points (D1 and D2): one to differentiate early caries (score, 1 vs. 2 or 3) and the other to differentiate severe caries requiring treatment (score, 1 or 2 vs. 3). The significance cut-off for all statistical tests was set at α = 0.05, using IBM SPSS Statistics for Windows, version 23.0 (IBM Corp., Armonk, NY, USA).

## Supplementary Information

Supplementary Figures.
