# Supplementary material for: Evaluation of dental caries detection with quantitative light-induced fluorescence in comparison to different field of view devices
Source: Sci Rep. 2022 Apr 12;12:6139. doi: 10.1038/s41598-022-10126-x (PMC9005513; doi:10.1038/s41598-022-10126-x)
Supplement: Supplementary file 1 — Supplementary Figures. [file 41598_2022_10126_MOESM1_ESM.pdf]

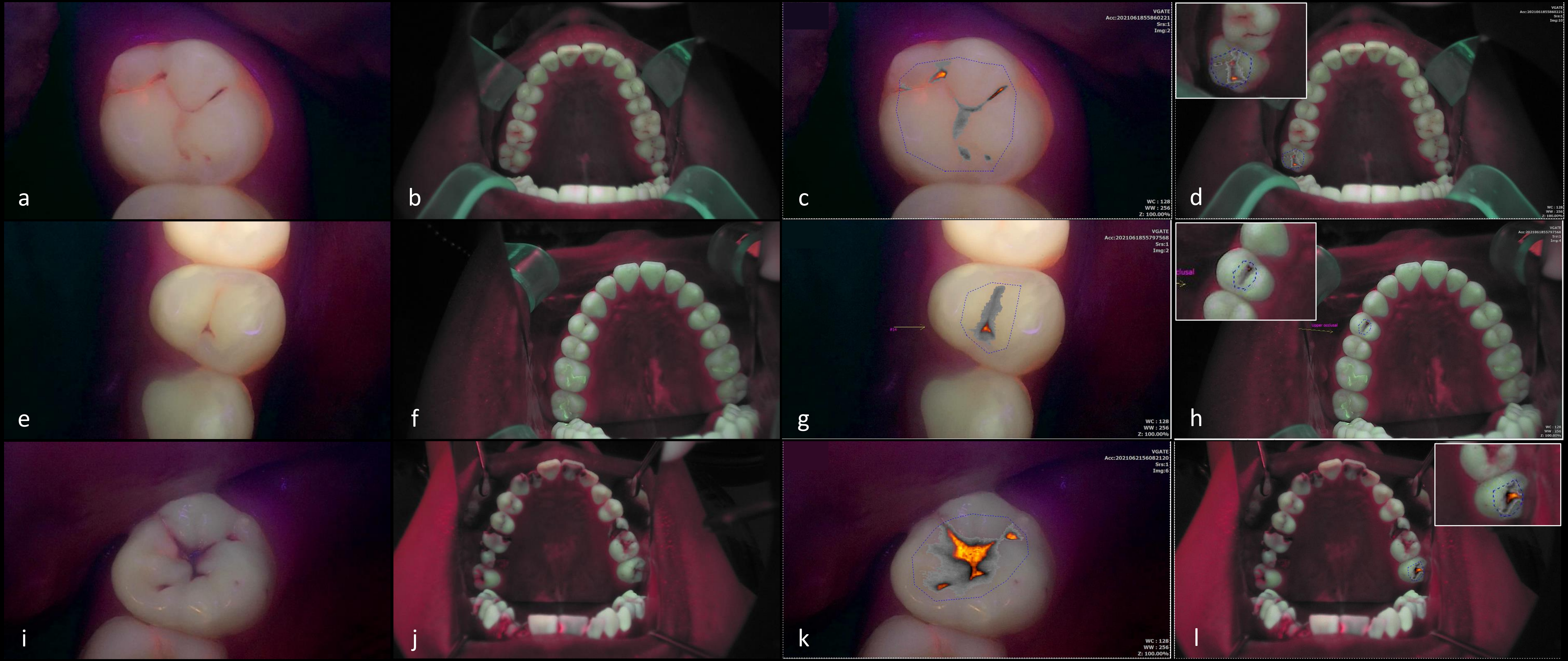

Figure 2A. Occlusal dental caries according to ICDAS II (International Caries Detection and Assessment System) Criteria: (a-d) Score 1 (first visual change in enamel) on maxillary right second molar (#17); (e-h) Score 2 (distinct visual change in enamel when viewed wet) on maxillary right first premolar (#14); Score 3 (localized enamel breakdown) on maxillary left second molar (#27); (a, e, i) Fluorescence image of Qraypen C; (b, f, j) Fluorescence image of Qraycam Pro; (c, g, k) Quantitative analysis of Qraypen C image using QA2 program; (d, h, l) Quantitative analysis of Qraycam Pro image using QA2 program.

Occlusal dental caries according to ICDAS II Criteria: Score 1 (first visual change in enamel) on maxillary right second molar (#17);
a) White image of Qraycam Pro; b) Fluorescence image of Qraycam Pro; c) Quantitative analysis of Qraycam Pro image using QA2 program.

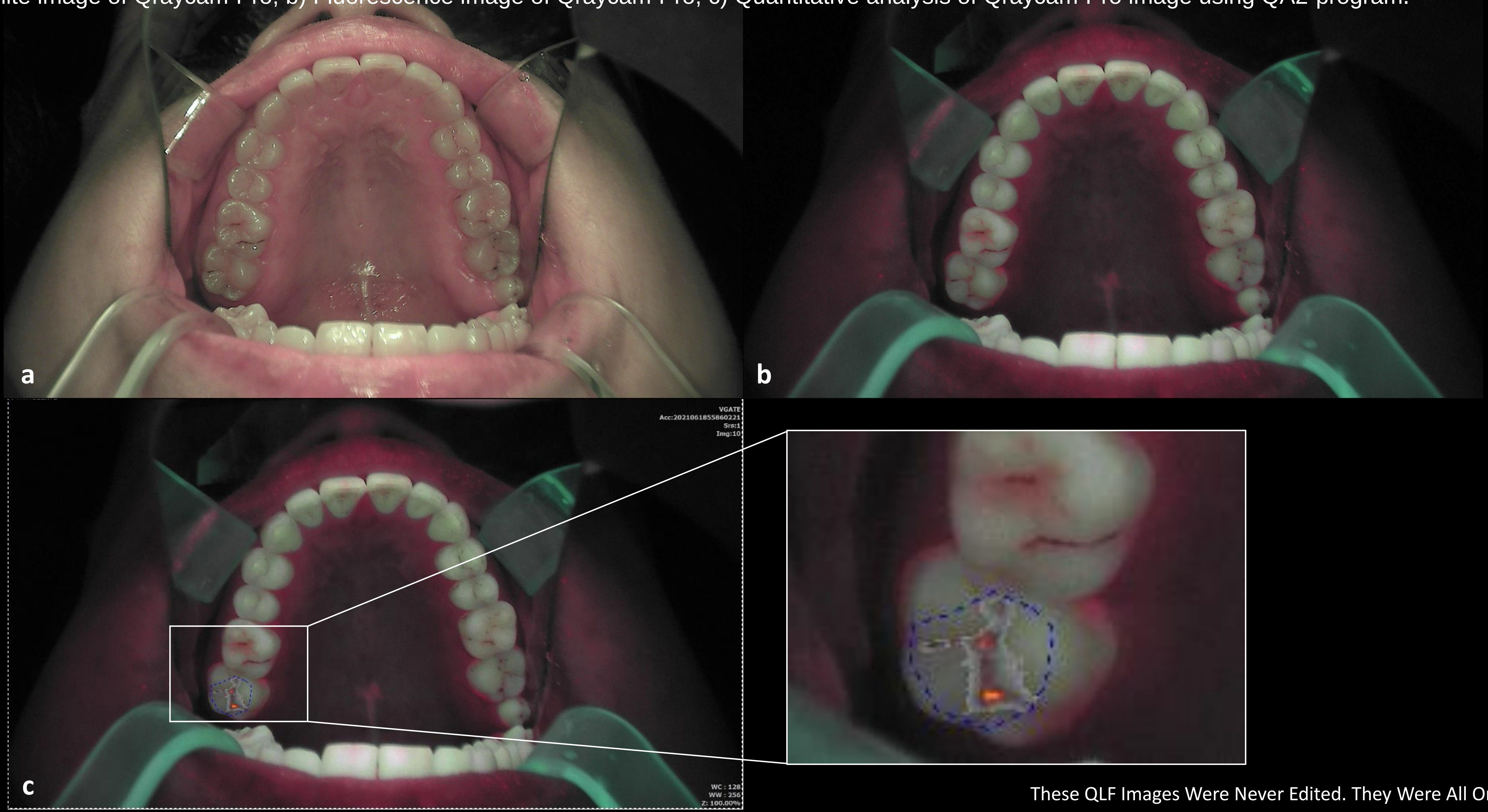

These QLF Images Were Never Edited. They Were All Original.

Occlusal dental caries according to ICDAS II Criteria: Score 1 (first visual change in enamel) on maxillary right second molar (#17);
a) White image of Qraypen C; b) Fluorescence image of Qraypen C; c) Quantitative analysis of Qraypen C image using QA2 program.

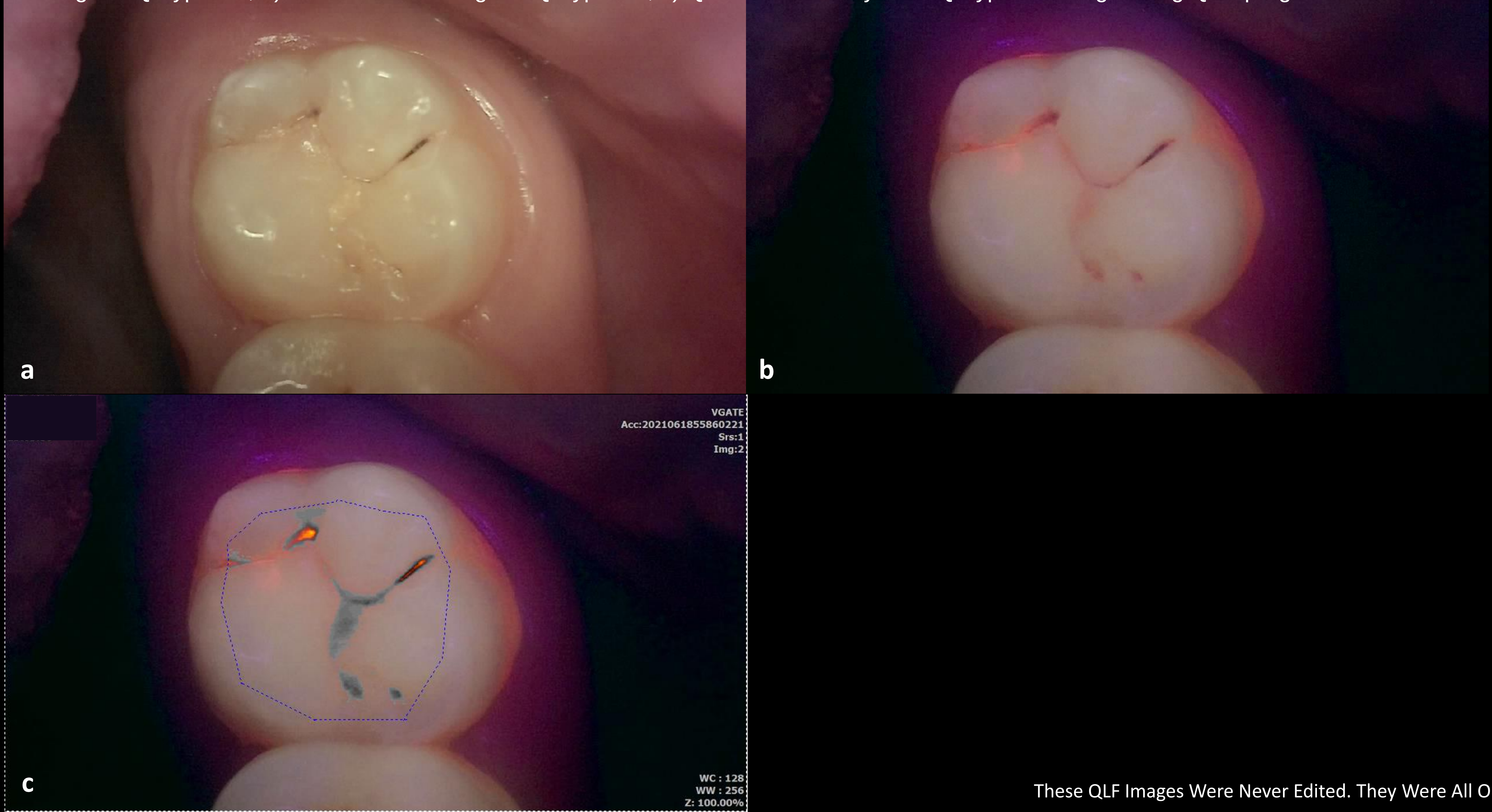

These QLF Images Were Never Edited. They Were All Original.

Occlusal dental caries according to ICDAS II Criteria: Score 2 (distinct visual change in enamel when viewed wet) on maxillary right first premolar (#14); a) White image of Qraycam Pro; b) Fluorescence image of Qraycam Pro; c) Quantitative analysis of Qraycam Pro image using QA2 program.

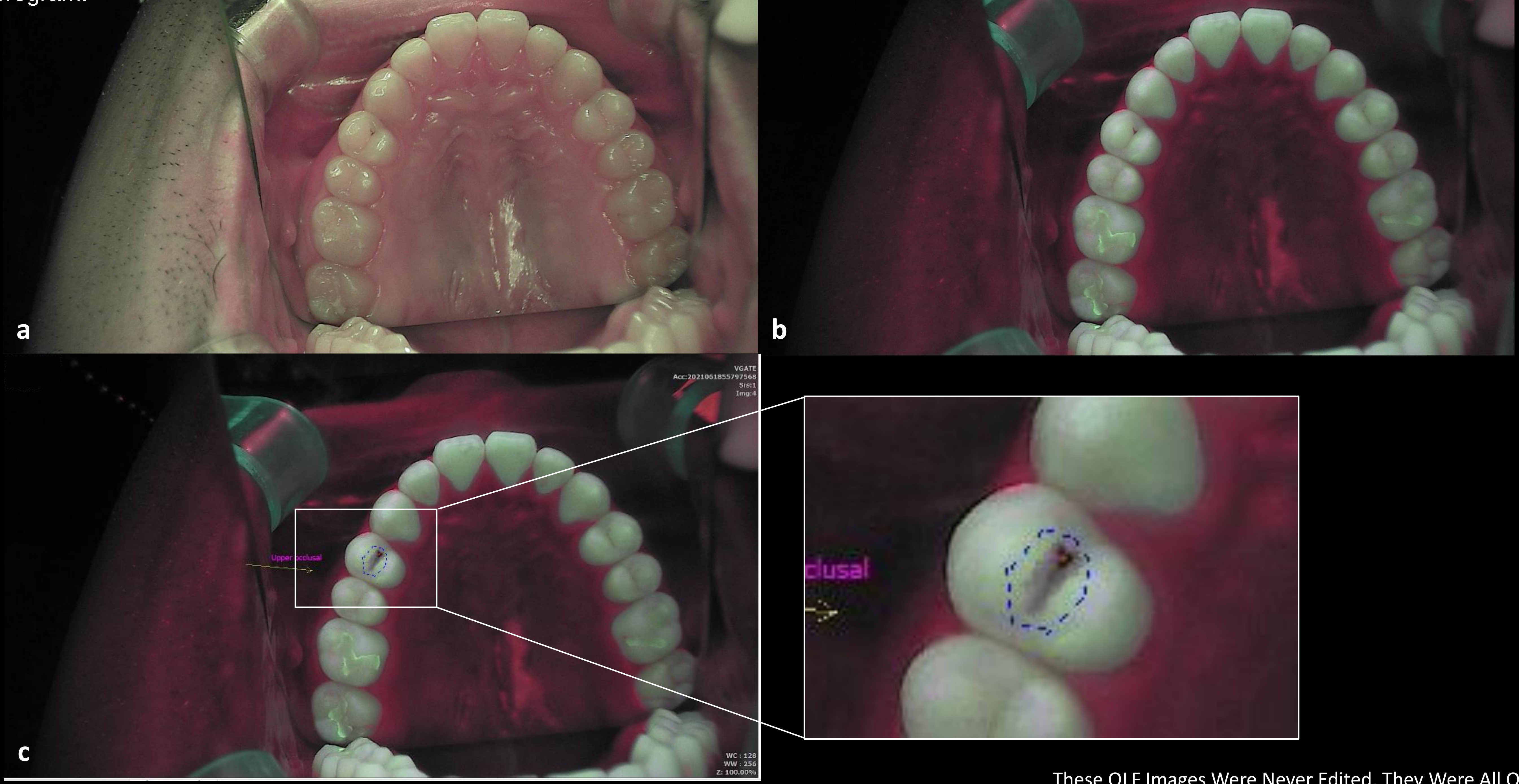

These QLF Images Were Never Edited. They Were All Original.

Occlusal dental caries according to ICDAS II Criteria: Score 2 (distinct visual change in enamel when viewed wet) on maxillary right first premolar (#14); a) White image of Qraypen C; b) Fluorescence image of Qraypen C; c) Quantitative analysis of Qraypen C image using QA2 program.

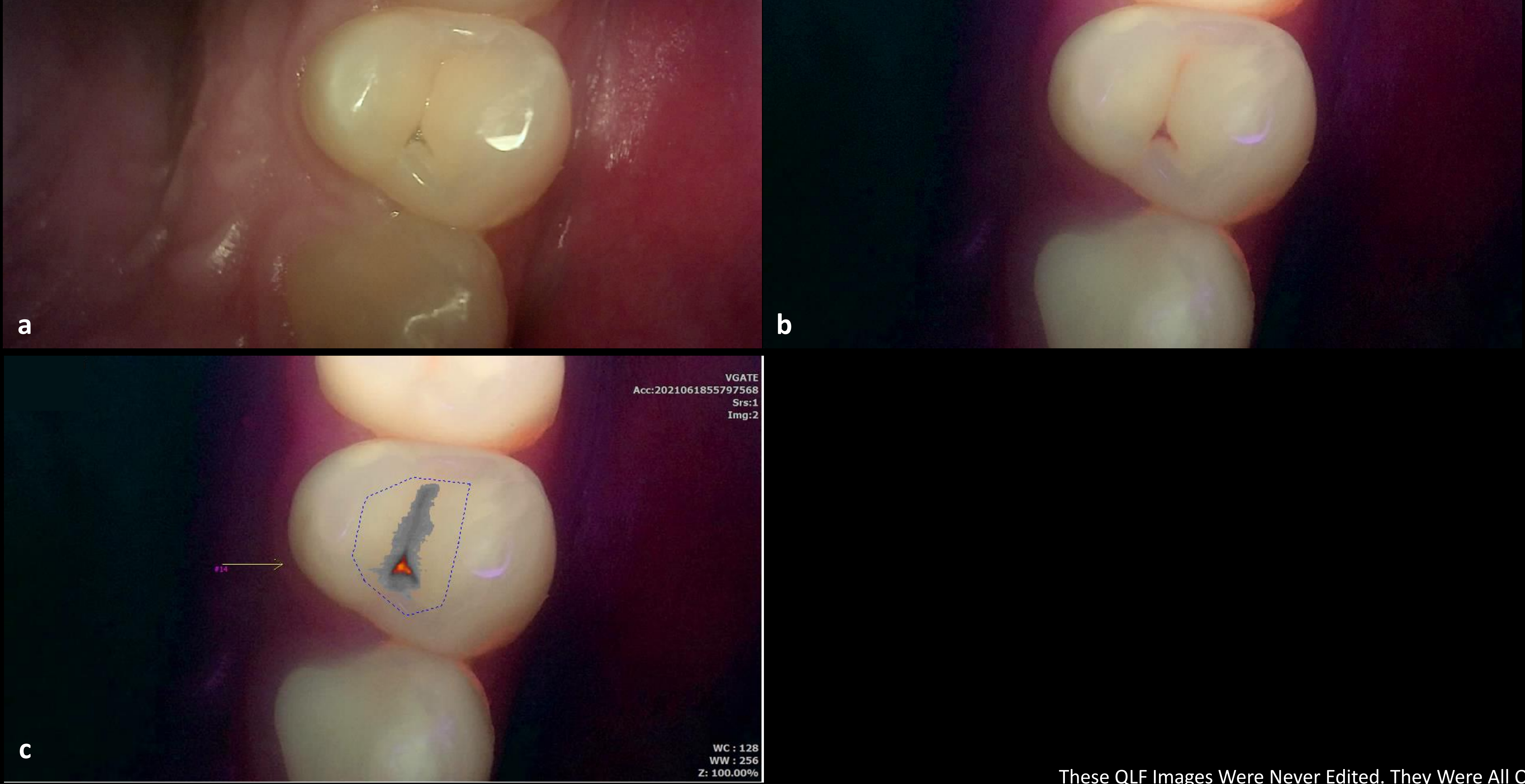

These QLF Images Were Never Edited. They Were All Original.

Occlusal dental caries according to ICDAS II Criteria: Score 3 (localized enamel breakdown) on maxillary left second molar (#27);
a) White image of Qraycam Pro; b) Fluorescence image of Qraycam Pro; c) Quantitative analysis of Qraycam Pro image using QA2 program.

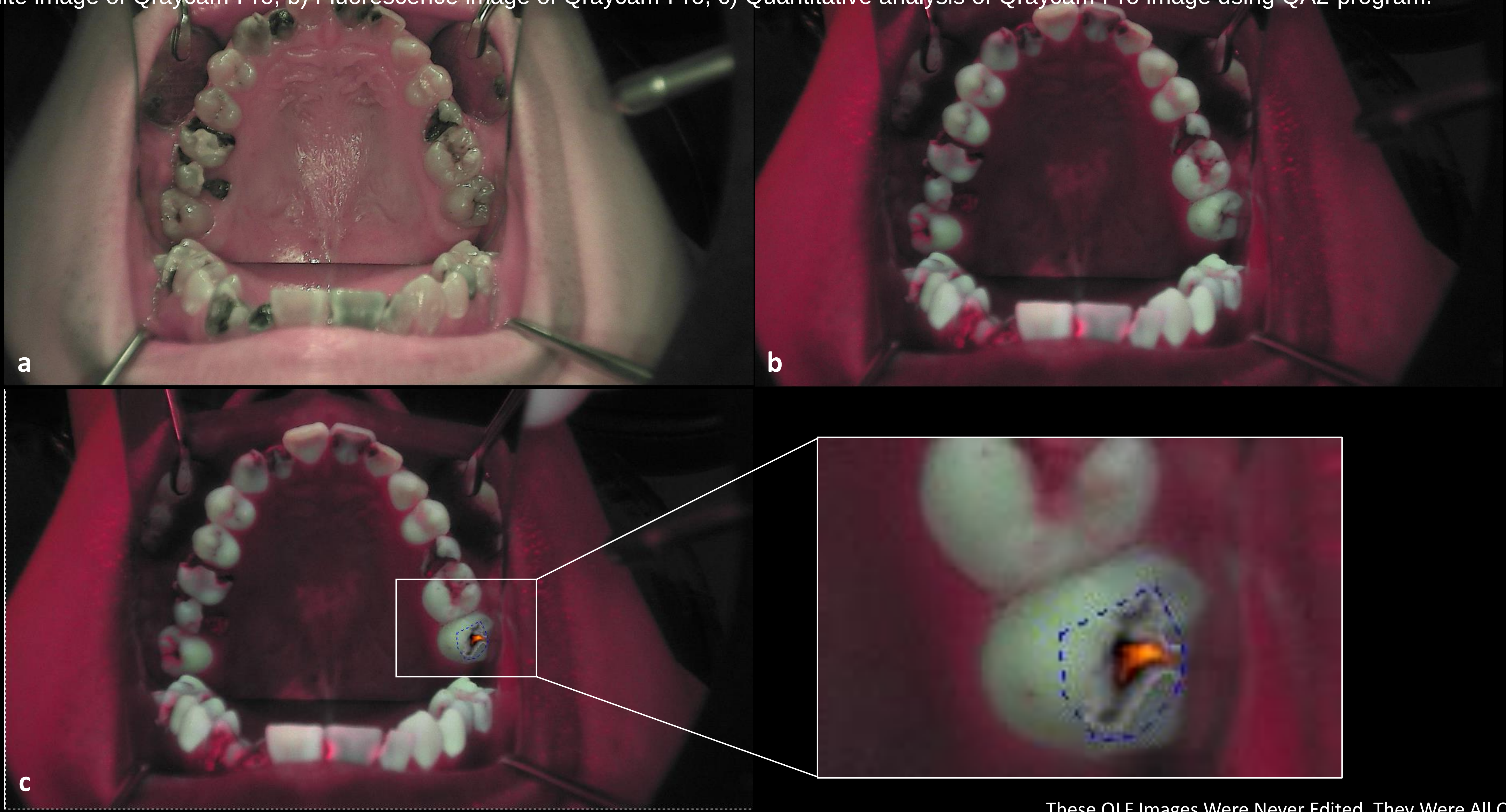

These QLF Images Were Never Edited. They Were All Original.

Occlusal dental caries according to ICDAS II Criteria:Score 3 (localized enamel breakdown) on maxillary left second molar (#27);
a) White image of Qraypen C; b) Fluorescence image of Qraypen C; c) Quantitative analysis of Qraypen C image using QA2 program.

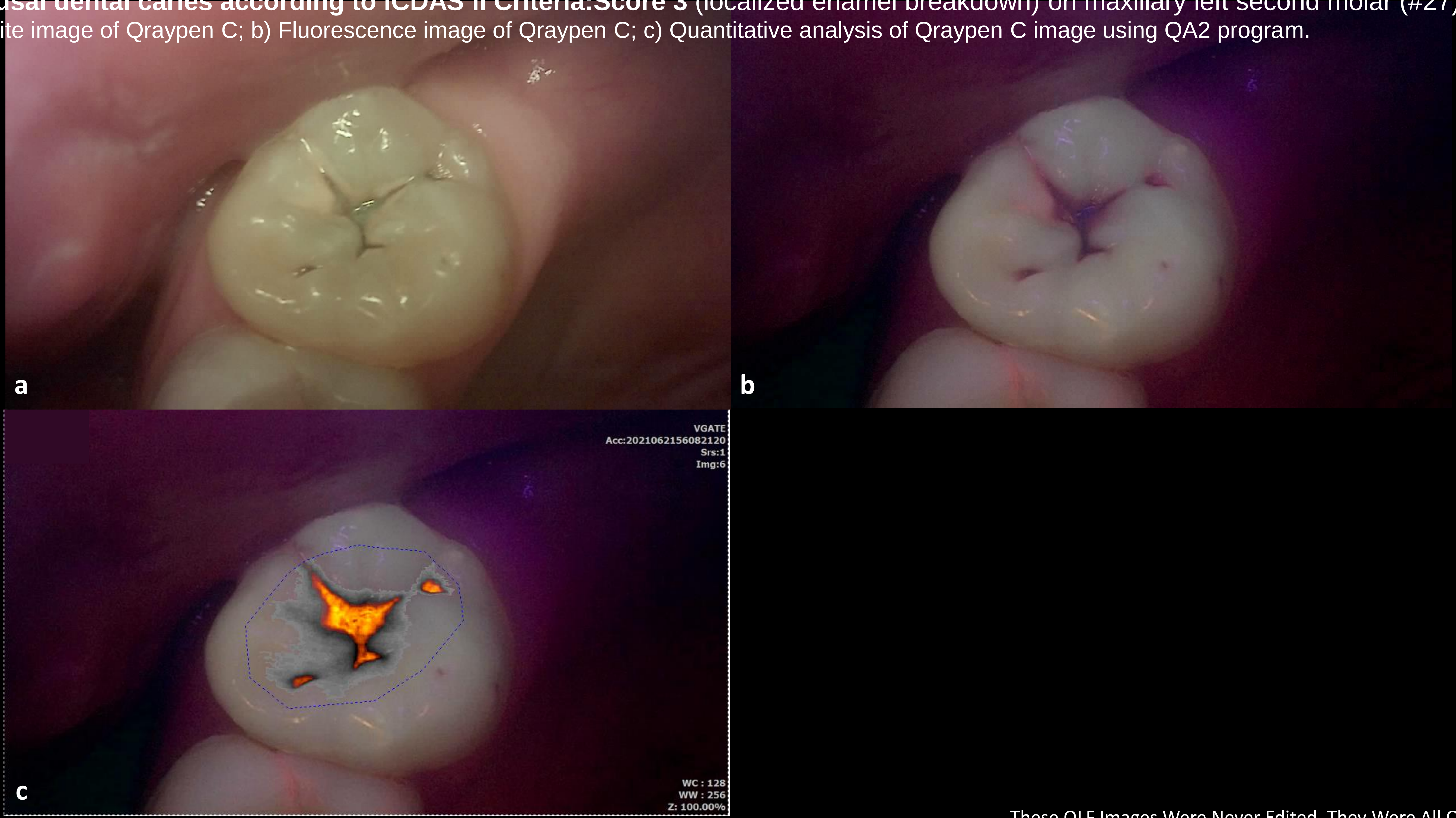

These QLF Images Were Never Edited. They Were All Original.

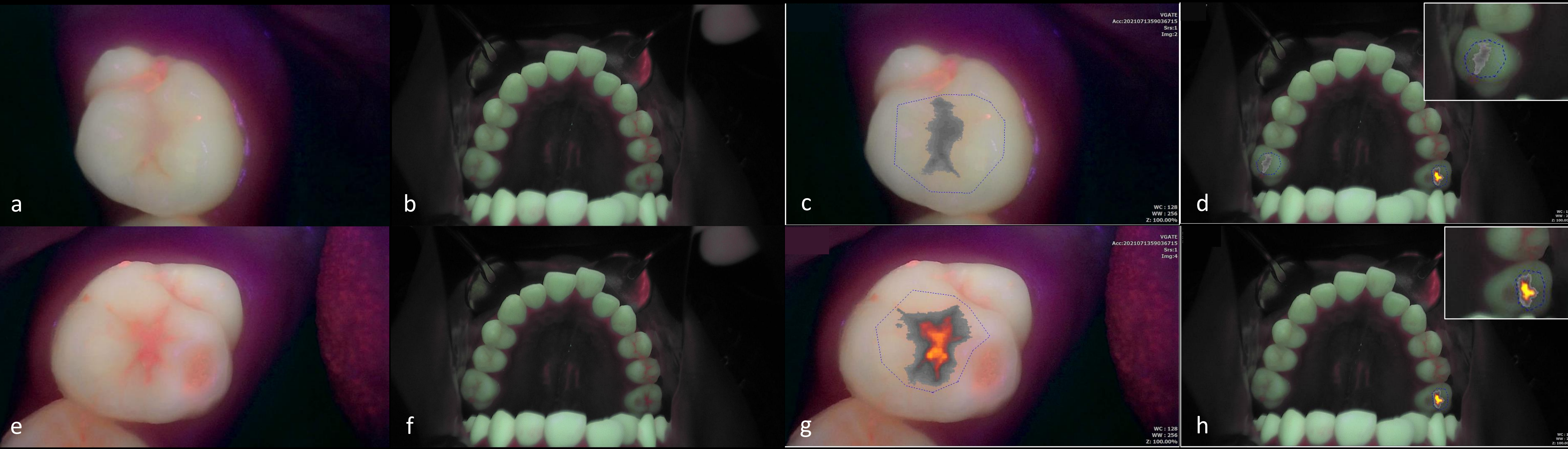

Figure 2B. Secondary dental caries according to ICDAS II Criteria: (a-d) Score 1 (first visual change in enamel) on maxillary left second molar (#27); (e-h) Score 2 (distinct visual change in enamel when viewed wet) on maxillary left second molar (#27); (a, e) Fluorescence image of Qraypen C; (b, f) Fluorescence image of Qraycam Pro; (c, g) Quantitative analysis of Qraypen C image using QA2 program; (d, h) Quantitative analysis of Qraycam Pro image using QA2 program.

Secondary dental caries according to ICDAS II Criteria: Score 1 (first visual change in enamel) on maxillary left second molar (#27);
a) White image of Qraycam Pro; b) Fluorescence image of Qraycam Pro; c) Quantitative analysis of Qraycam Pro image using QA2 program.

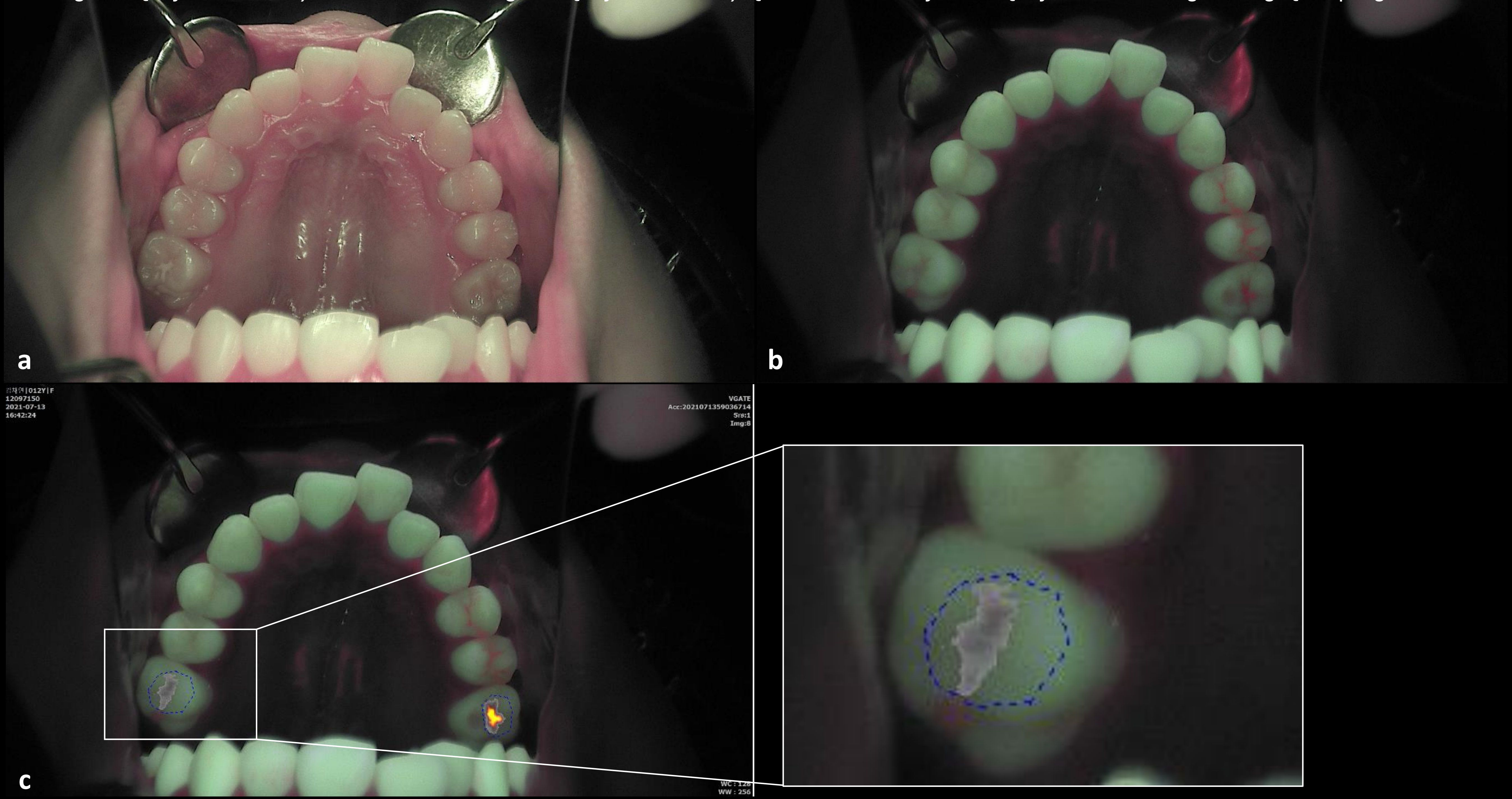

These QLF Images Were Never Edited. They Were All Original.

Secondary dental caries according to ICDAS II Criteria: Score 1 (first visual change in enamel) on maxillary left second molar (#27);
a) White image of Qraypen C; b) Fluorescence image of Qraypen C; c) Quantitative analysis of Qraypen C image using QA2 program.

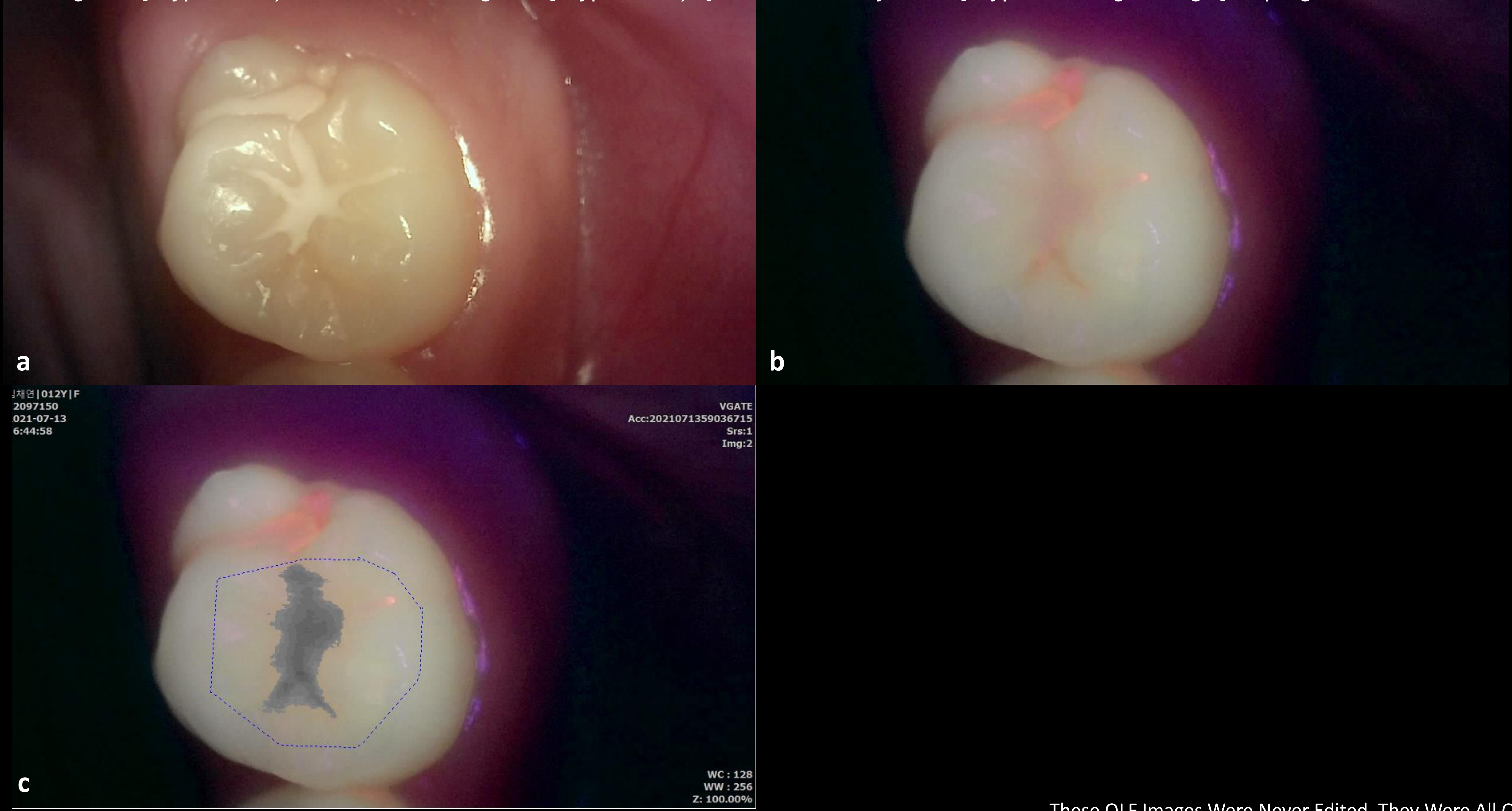

These QLF Images Were Never Edited. They Were All Original.

Secondary dental caries according to ICDAS II Criteria: Score 2 (distinct visual change in enamel when viewed wet) on maxillary left second molar (#27); a) White image of Qraycam Pro; b) Fluorescence image of Qraycam Pro; c) Quantitative analysis of Qraycam Pro image using QA2 program.

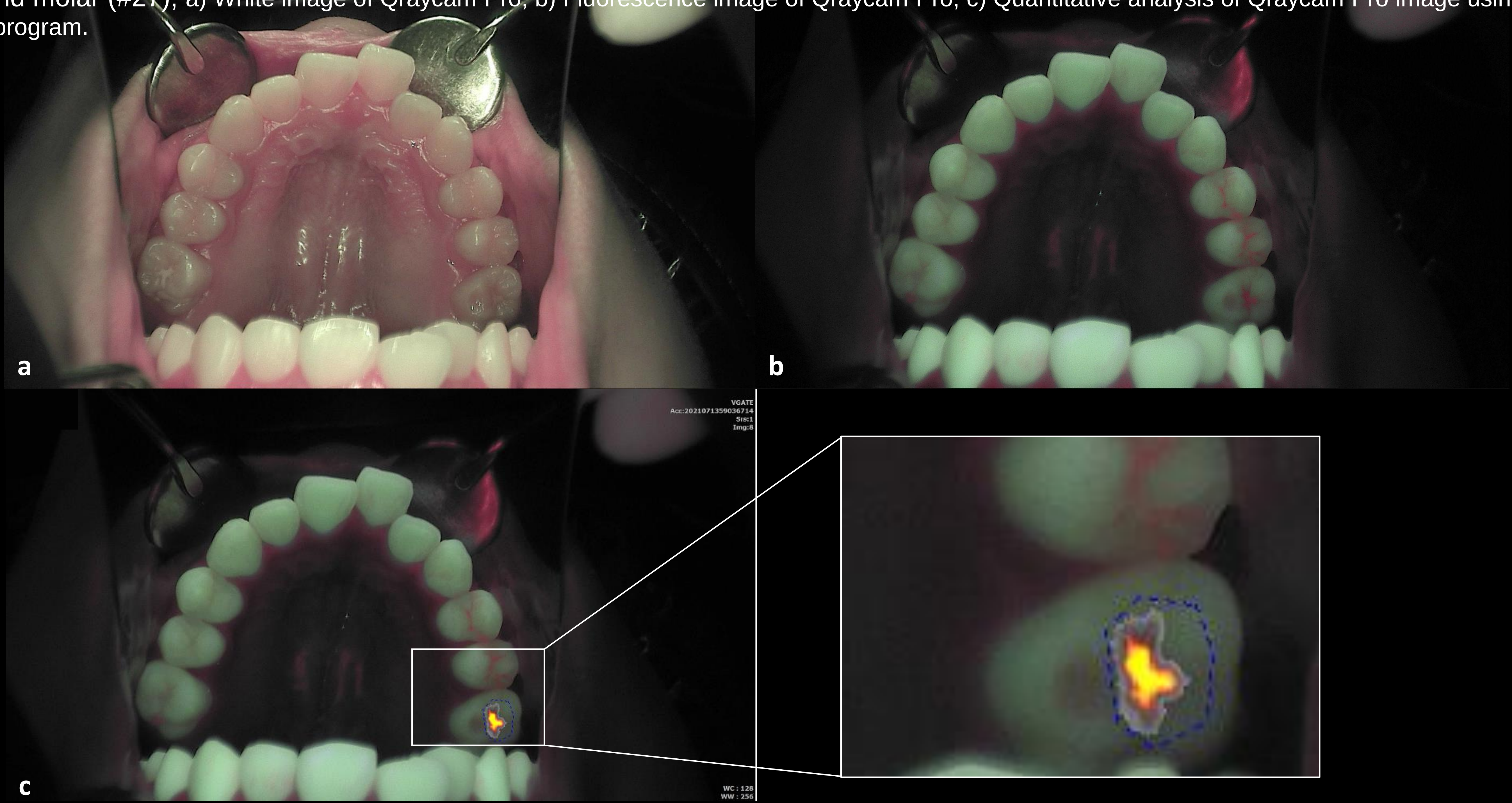

These QLF Images Were Never Edited. They Were All Original.

Secondary dental caries according to ICDAS II Criteria: Score 2 (distinct visual change in enamel when viewed wet) on maxillary left second molar (#27); a) White image of Qraypen C; b) Fluorescence image of Qraypen C; c) Quantitative analysis of Qraypen C image using QA2 program.

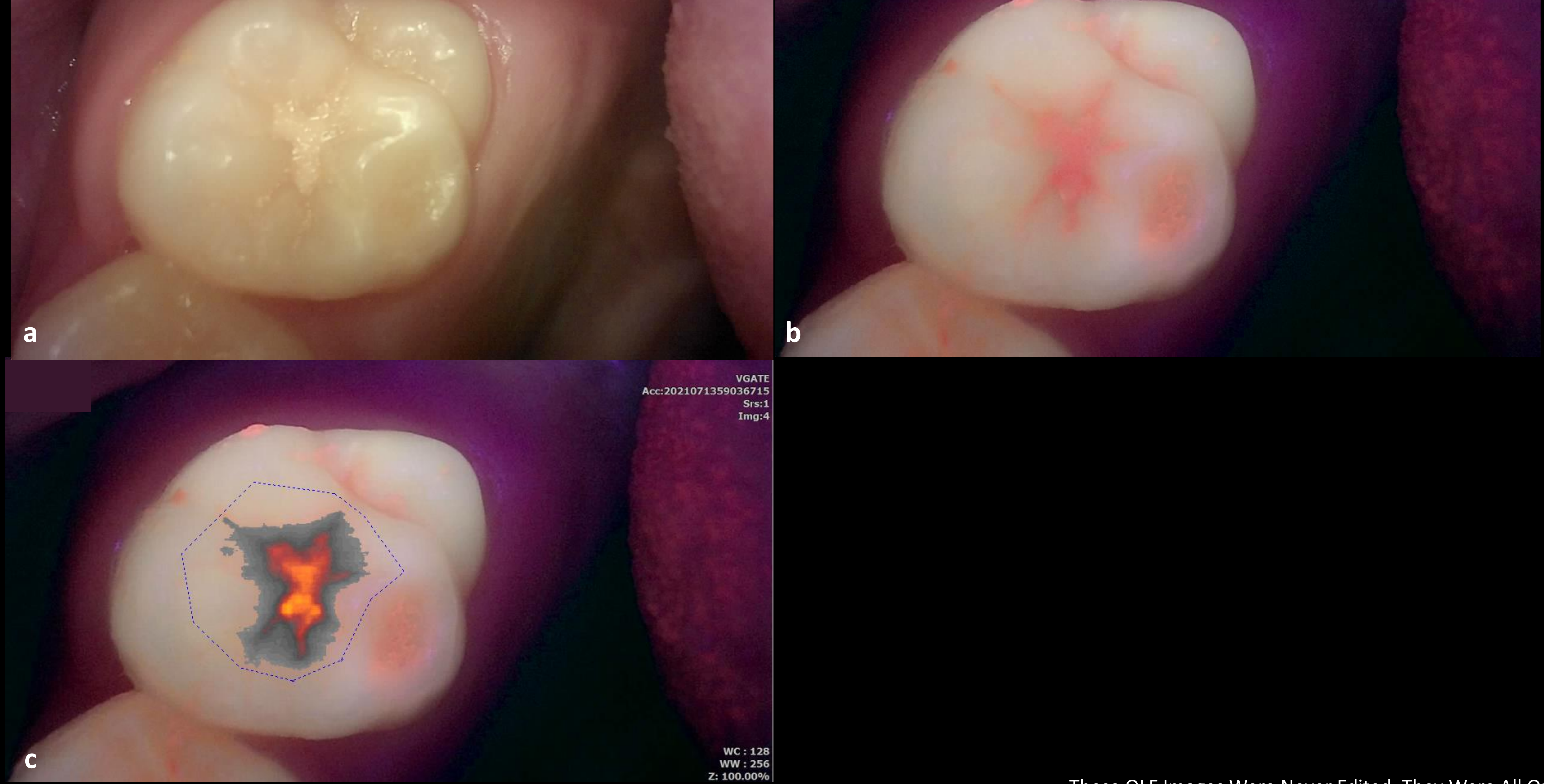

These QLF Images Were Never Edited. They Were All Original.

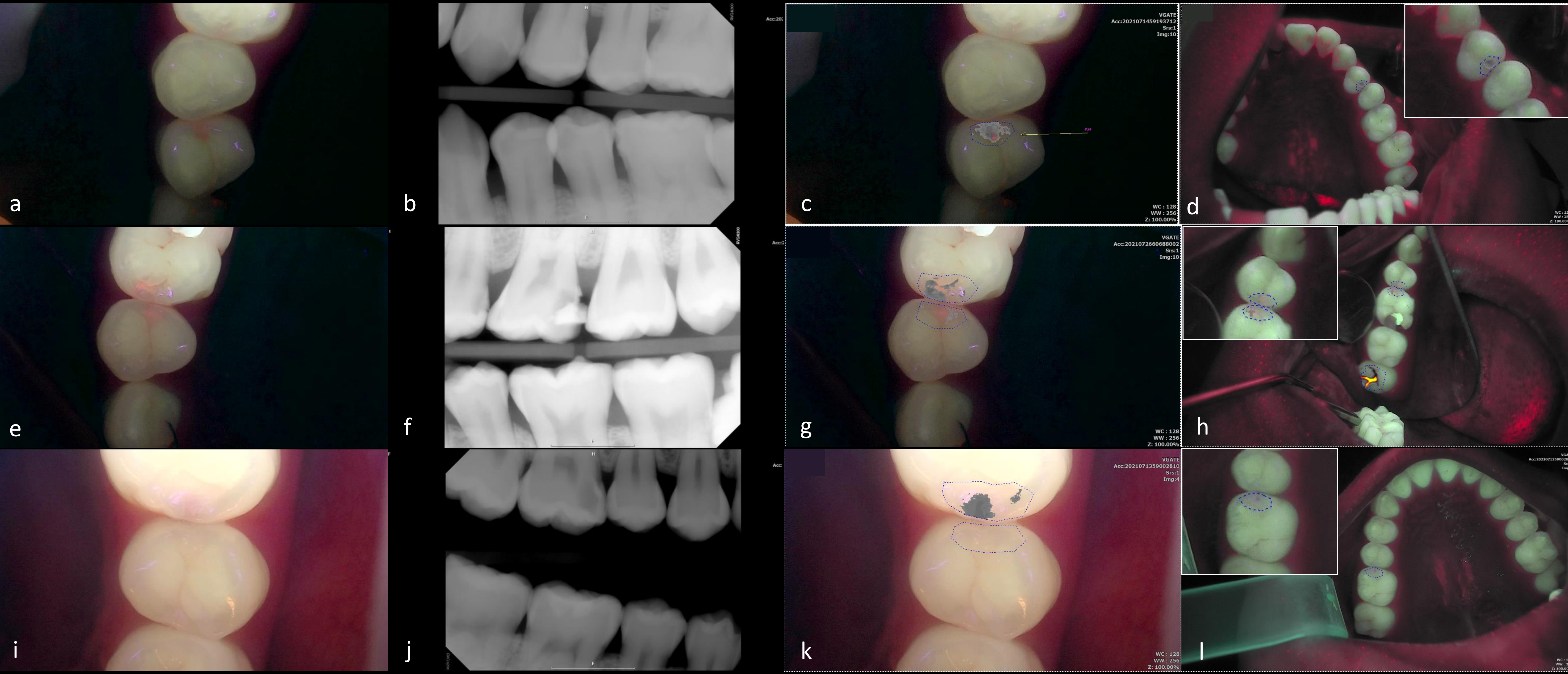

Figure 2C. Proximal dental caries according to X-ray Criteria: (a-d) Score 1 (radiolucency visible in the enamel) on maxillary left first premolar (#24, arrow) (e-h) Score 2 (radiolucency in the dentin but restricted to the outer 1/3 of the dentin) on maxillary left second premolar (#25, arrow); Score 3 (radiolucency extending to the middle 1/3 of the dentin) on maxillary right first molar (#16, arrow); (a, e, i) Fluorescence image of Qraypen C; (b, f, j) bitewing radiograph; (c, g, k) Quantitative analysis of Qraypen C image using QA2 program; (d, h, l) Quantitative analysis of Qraycam Pro image using QA2 program.

Proximal dental caries according to X-ray Criteria: Score 1 (radiolucency visible in the enamel) on maxillary left first premolar (#24, distal); a) White image of Qraycam Pro; b) Fluorescence image of Qraycam Pro; c) Quantitative analysis of Qraycam Pro image using QA2 program.

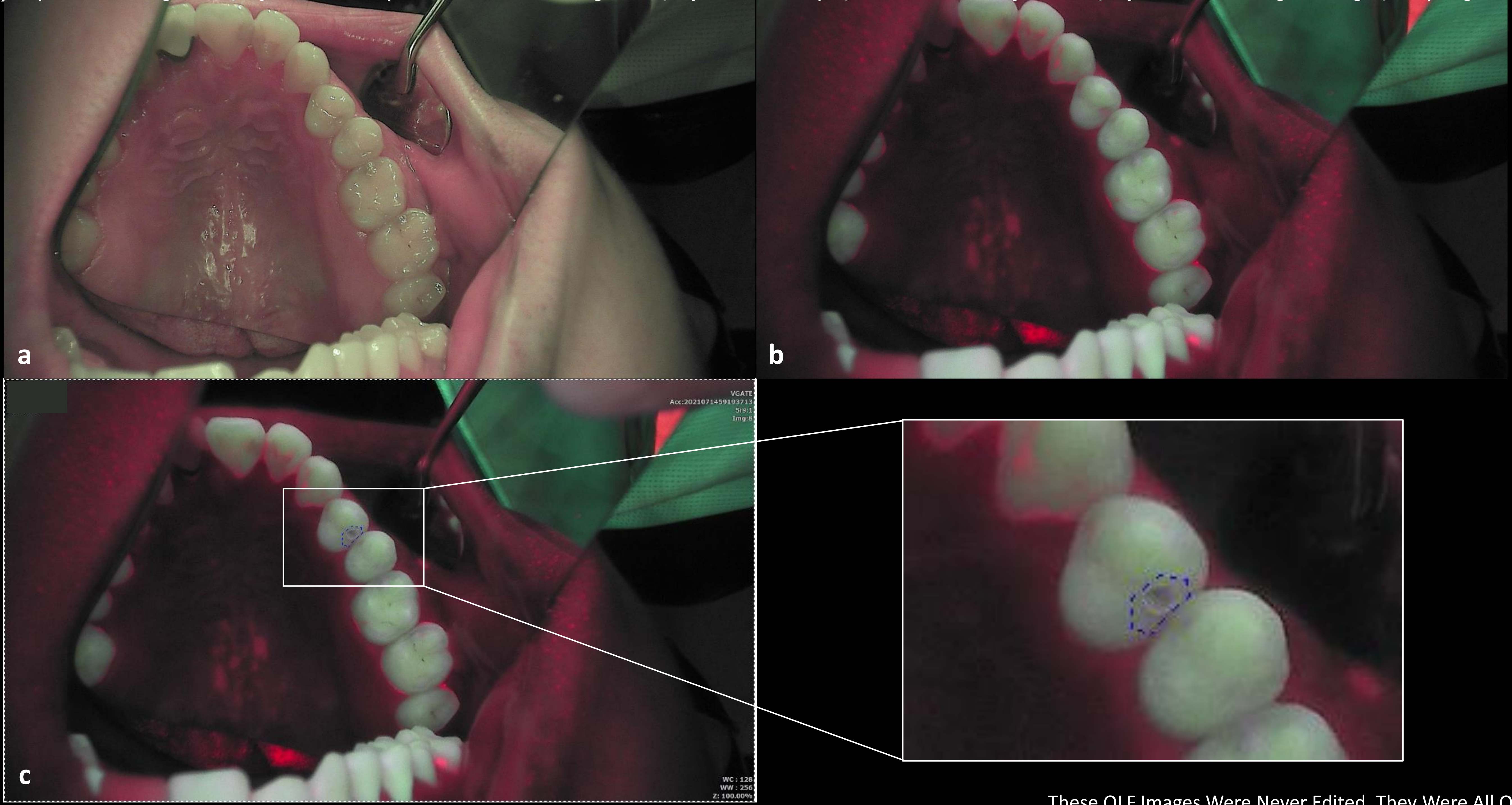

These QLF Images Were Never Edited. They Were All Original.

Proximal dental caries according to X-ray Criteria: Score 1 (radiolucency visible in the enamel) on maxillary left first premolar (#24, distal); a) White image of Qraypen C; b) Fluorescence image of Qraypen C; c) Quantitative analysis of Qraypen C image using QA2 program; d) Bitewing radiograph.

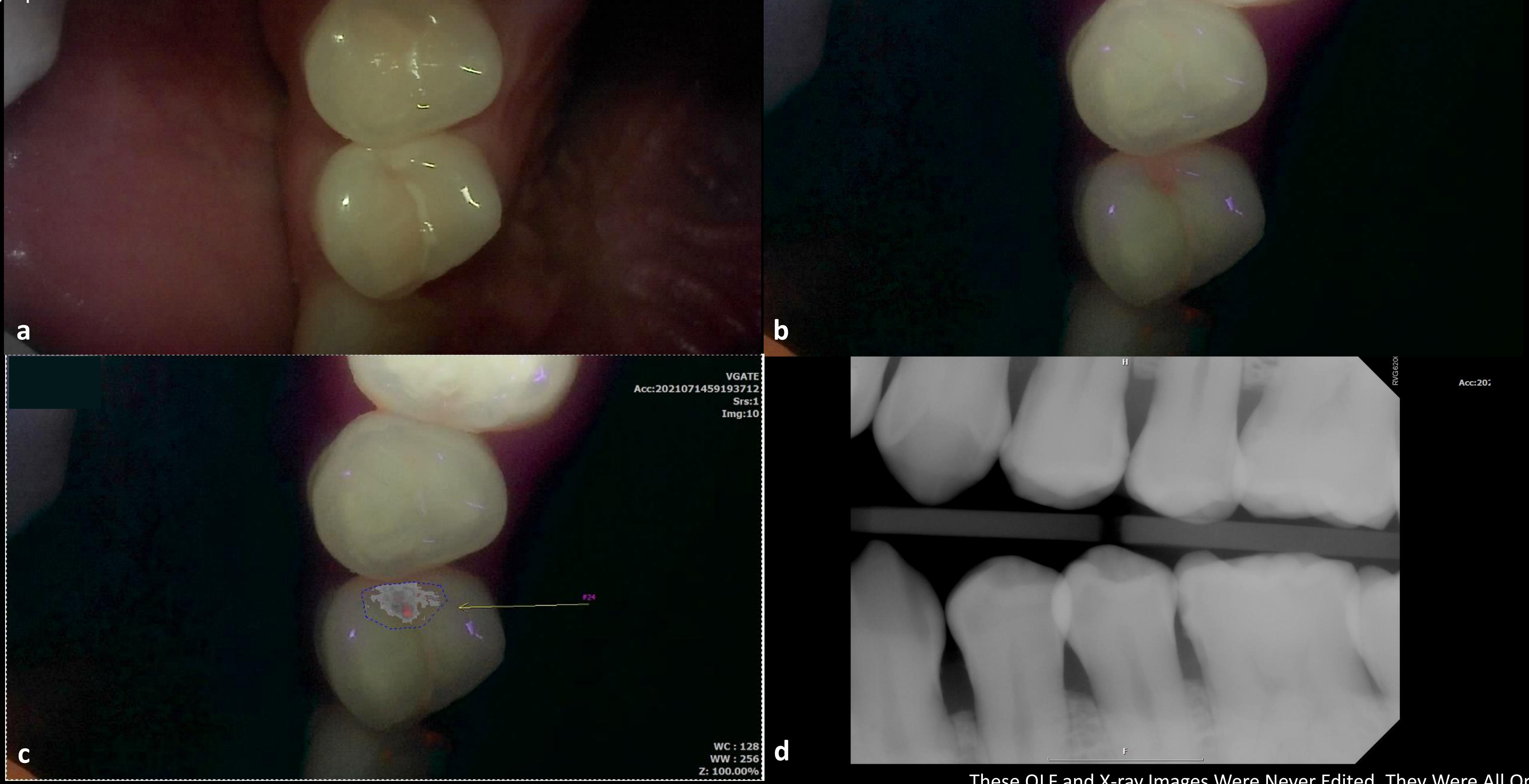

These QLF and X-ray Images Were Never Edited. They Were All Original.

Proximal dental caries according to X-ray Criteria: Score 2 (radiolucency in the dentin but restricted to the outer 1/3 of the dentin) on maxillary left second premolar (#25, distal); a) White image of Qraycam Pro; b) Fluorescence image of Qraycam Pro; c) Quantitative analysis of Qraycam Pro image using QA2 program.

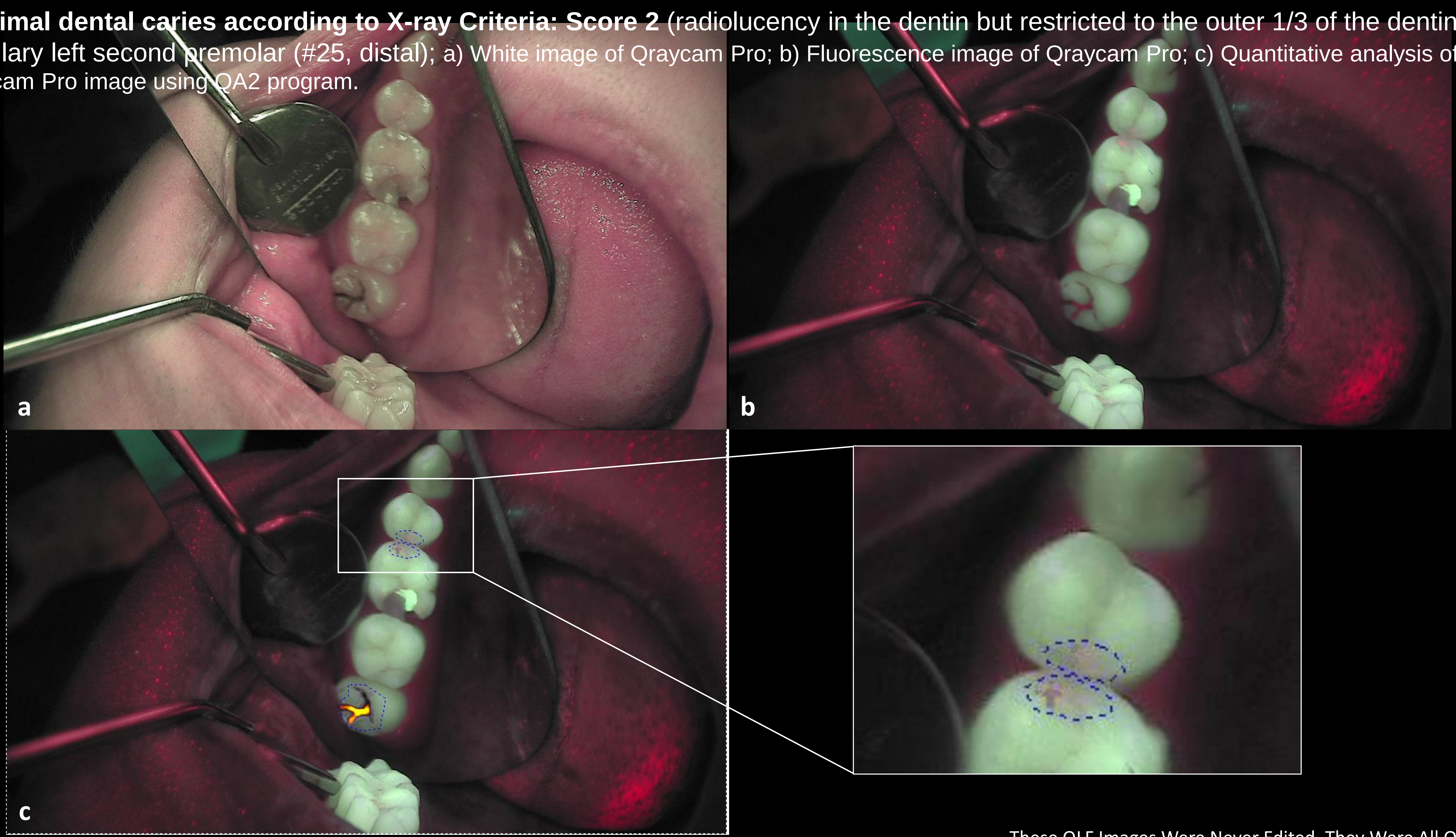

These QLF Images Were Never Edited. They Were All Original.

Proximal dental caries according to X-ray Criteria: Score 2 (radiolucency in the dentin but restricted to the outer 1/3 of the dentin) on maxillary left second premolar (#25, distal); a) White image of Qraypen C; b) Fluorescence image of Qraypen C; c) Quantitative analysis of Qraypen C image using QA2 program; d) Bitewing radiograph.

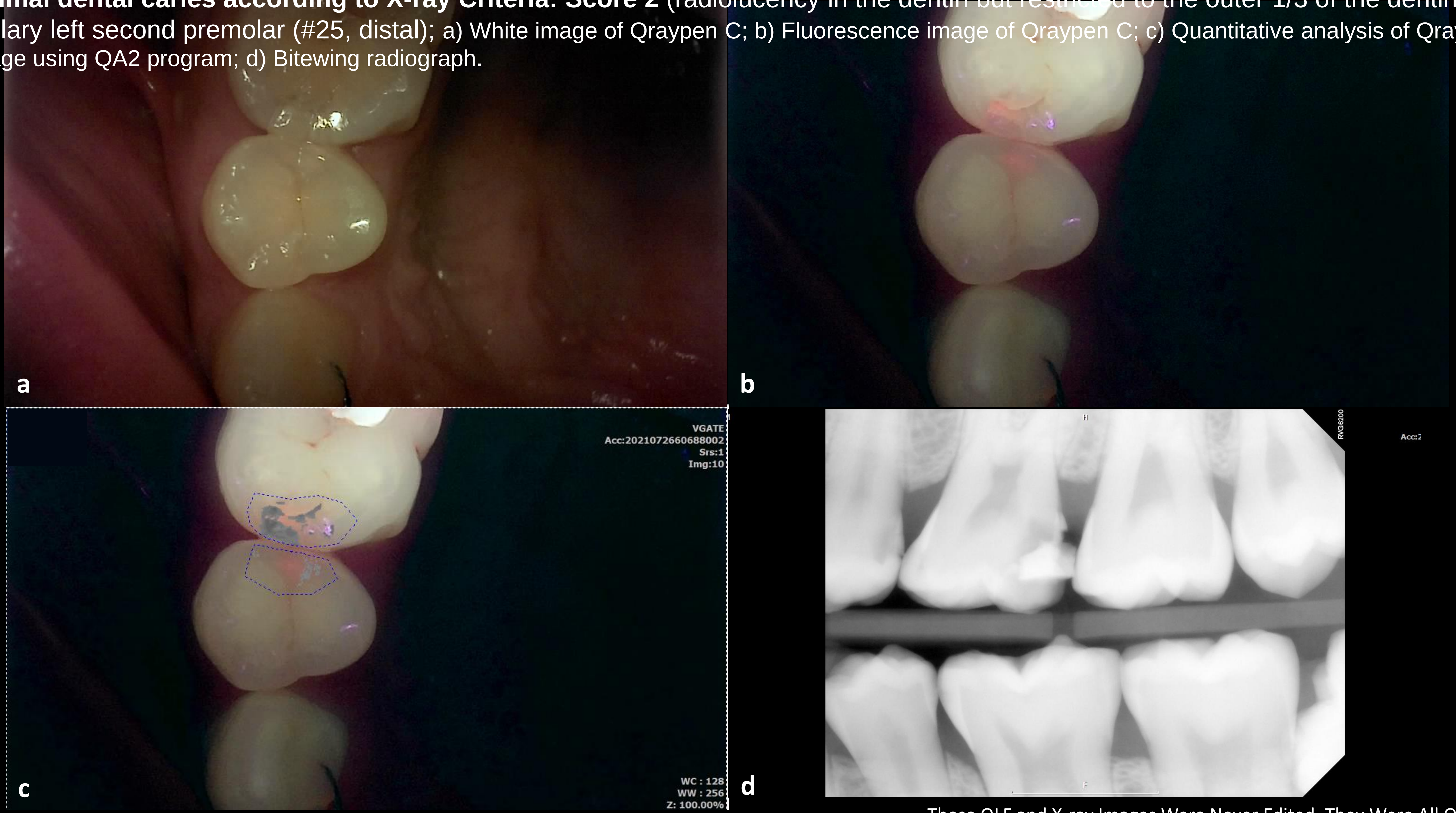

These QLF and X-ray Images Were Never Edited. They Were All Original.

Proximal dental caries according to X-ray Criteria: Score 3 (radiolucency extending to the middle 1/3 of the dentin) on maxillary right first molar (#16, mesial); a) White image of Qraycam Pro; b) Fluorescence image of Qraycam Pro; c) Quantitative analysis of Qraycam Pro image using QA2 program.

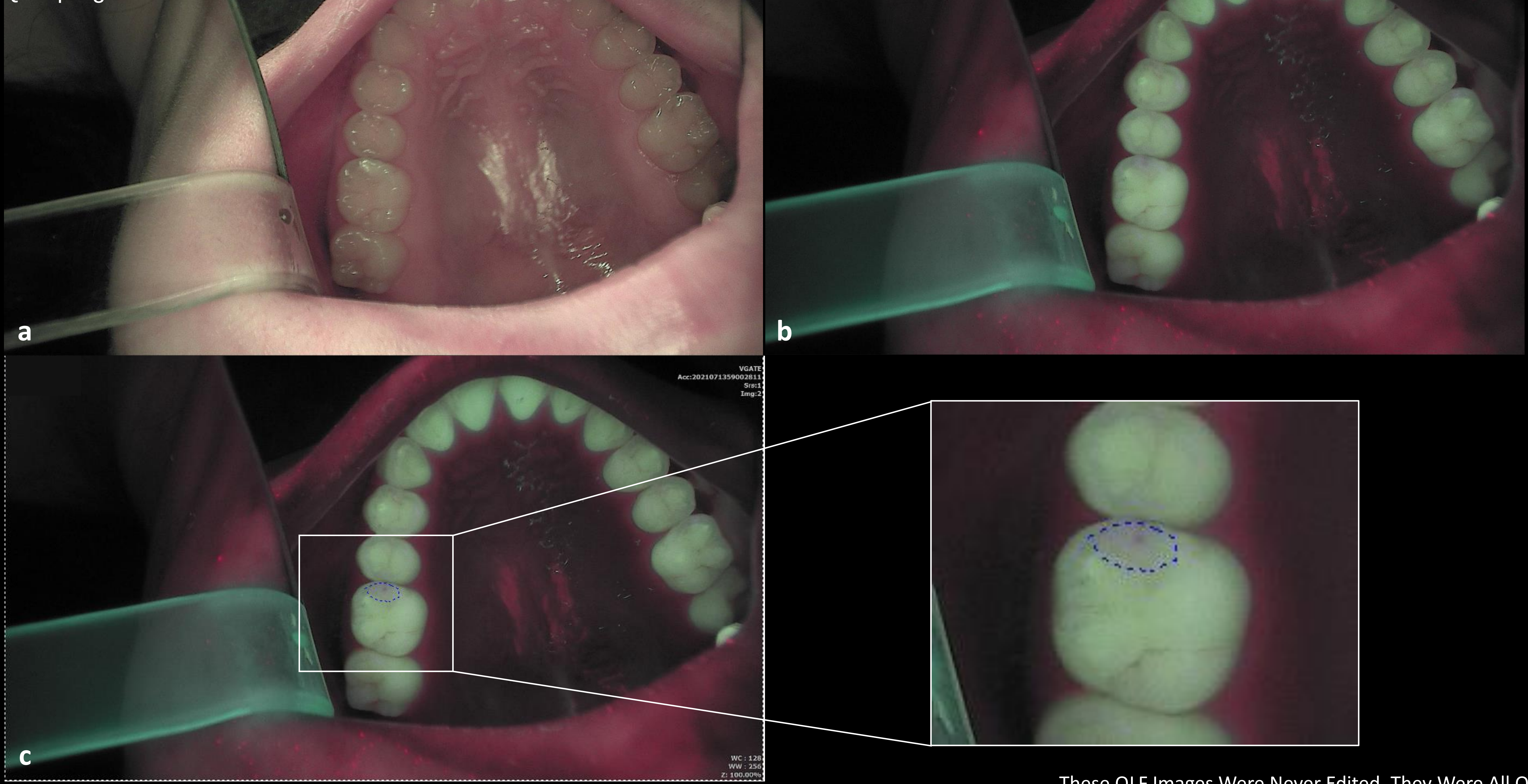

These QLF Images Were Never Edited. They Were All Original.

Proximal dental caries according to X-ray Criteria: Score 3 (radiolucency extending to the middle 1/3 of the dentin) on maxillary right first molar (#16, mesial); a) White image of Qraypen C; b) Fluorescence image of Qraypen C; c) Quantitative analysis of Qraypen C image using QA2 program; d) Bitewing radiograph.

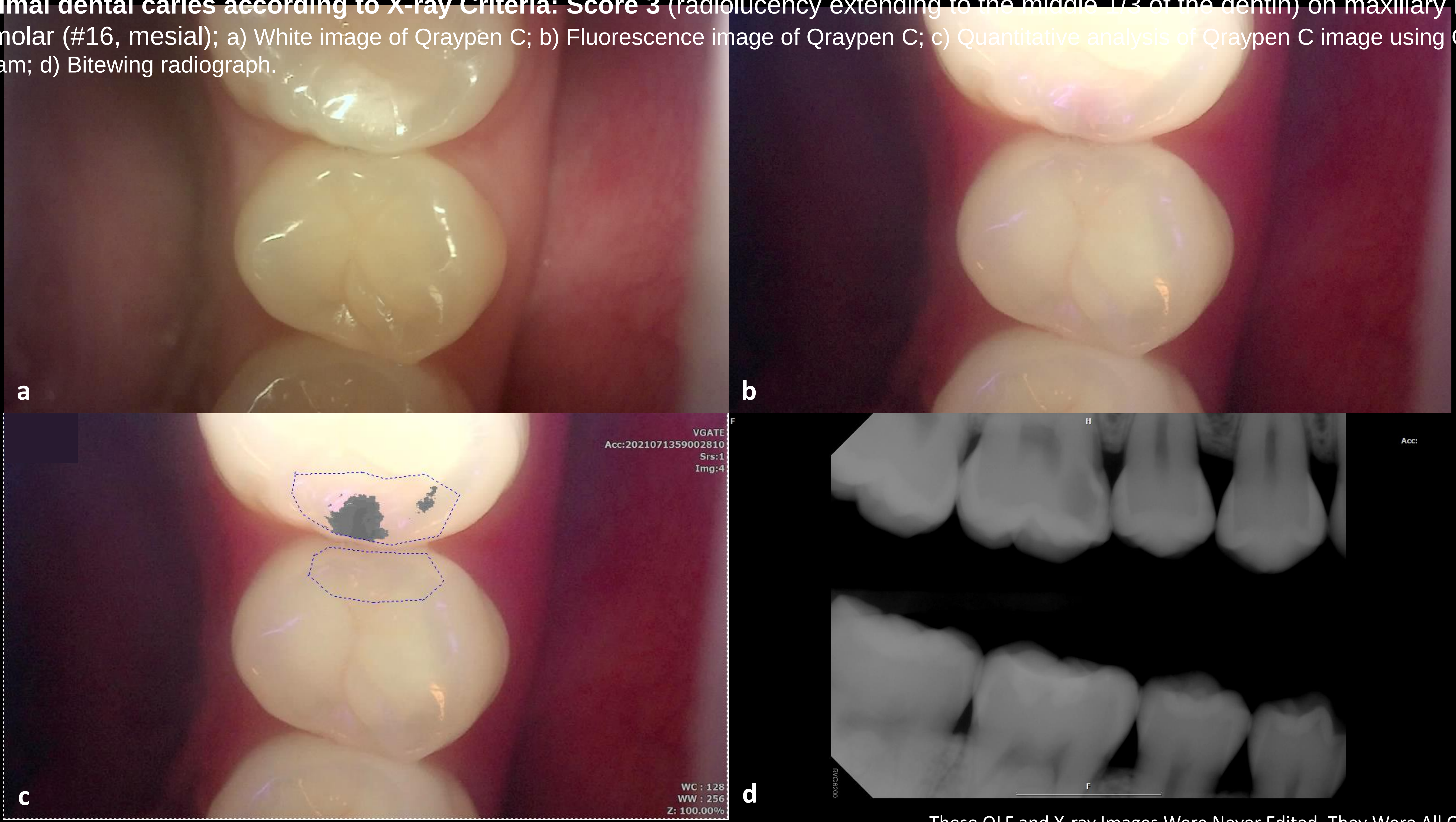

These QLF and X-ray Images Were Never Edited. They Were All Original.
